# M1-like monocytes are a major immunological determinant of severity in previously healthy adults with life-threatening influenza

**DOI:** 10.1172/jci.insight.91868

**Published:** 2017-04-06

**Authors:** Suzanne L. Cole, Jake Dunning, Wai Ling Kok, Kambez Hajipouran Benam, Adel Benlahrech, Emmanouela Repapi, Fernando O. Martinez, Lydia Drumright, Timothy J. Powell, Michael Bennett, Ruth Elderfield, Catherine Thomas, MOSAIC investigators, Tao Dong, John McCauley, Foo Y. Liew, Stephen Taylor, Maria Zambon, Wendy Barclay, Vincenzo Cerundolo, Peter J. Openshaw, Andrew J. McMichael, Ling-Pei Ho

**Affiliations:** 1 MRC Human Immunology Unit, Weatherall Institute of Molecular Medicine, University of Oxford, Oxford, United Kingdom.; 2 National Heart and Lung Division, Imperial College London, St. Mary’s Campus, London, United Kingdom.; 3 Computational Biology Group, Weatherall Institute of Molecular Medicine, University of Oxford, Oxford, United Kingdom.; 4 School of Biosciences and Medicine, University of Surrey, Guildford, United Kingdom.; 5 The Francis Crick Institute, London, United Kingdom.; 6 Section of Virology, Faculty of Medicine, Wright Fleming Institute, Imperial College London, London, United Kingdom.; 7 National Infection Service, Public Health England, Colindale, London, United Kingdom.; 8 The MOSAIC investigators are detailed in the Supplemental Acknowledgments.; 9 Division of Immunology, Infection and Inflammation, University of Glasgow, Glasgow, United Kingdom.; 10 School of Biology and Basic Medical Sciences, Soochow University, Suzhou, China.

## Abstract

In each influenza season, a distinct group of young, otherwise healthy individuals with no risk factors succumbs to life-threatening infection. To better understand the cause for this, we analyzed a broad range of immune responses in blood from a unique cohort of patients, comprising previously healthy individuals hospitalized with and without respiratory failure during one influenza season, and infected with one specific influenza A strain. This analysis was compared with similarly hospitalized influenza patients with known risk factors (total of *n* = 60 patients recruited). We found a sustained increase in a specific subset of proinflammatory monocytes, with high TNF-α expression and an M1-like phenotype (independent of viral titers), in these previously healthy patients with severe disease. The relationship between M1-like monocytes and immunopathology was strengthened using murine models of influenza, in which severe infection generated using different models (including the high-pathogenicity H5N1 strain) was also accompanied by high levels of circulating M1-like monocytes. Additionally, a raised M1/M2 macrophage ratio in the lungs was observed. These studies identify a specific subtype of monocytes as a modifiable immunological determinant of disease severity in this subgroup of severely ill, previously healthy patients, offering potential novel therapeutic avenues.

## Abstract 2

In a cohort of influenza patients, previously healthy and young patients who succumbed to life-threatening disease were defined by high levels of circulating M1-like, TNF-α^hi^ monocytes.

## Introduction

Seasonal influenza epidemics can affect 5%–10% of adults, causing up to 0.5 million deaths annually worldwide (1). The most severe disease occurs in those with diverse factors associated with a suboptimal immune response, for example, children under 5 years of age, the elderly, those who are pregnant or on immunosuppressants, or those with haematological malignancies. Every year, and particularly in novel outbreaks, there is also a small, distinct group of previously healthy young adults (less than 50 years old) who succumbs to severe disease to the point of requiring ventilatory support. Indeed, during the 2009 pandemic (pH1N1), although most individuals infected with pH1N1 influenza A virus (IAV) had mild or uncomplicated illness, there was a discrete prevalence of severe disease in otherwise healthy persons. In fact, 90% of all deaths in the pandemic occurred in those under 65 years of age (2–4). An “immune storm” has been suggested to underlie these severe cases, but the critical cellular immune cause remains unclear.

The mechanisms by which influenza infections cause severe disease are complex and often multifactorial, involving host and viral factors, and secondary bacterial infection (5–8). A cytokine-mediated inflammatory response has been shown to cause organ injury and influenza sepsis syndrome, and high levels of TNF-α, IL-6, KC, and MCP-1 are established causes of lung pathology in murine models (6). NK responses and neutrophils, both critical for immune protection, can also contribute to pulmonary inflammation during severe infection (9). Improper contraction or excessive production of TNF-α from antigen-specific CD8 T cells and high levels of antigen-specific CD4 T cells and IL-17 have also been shown to correlate with immunopathology (10, 11). However, the cause of severe infection, specifically in young previously healthy adults, remains unknown, in part because of their relative low frequency during an influenza season. In outbreaks involving novel strains of the virus, such as the H5N1 outbreak in 2010, younger adults were disproportionately affected and reported to have marked hypercytokinemia (12). However, it is unclear whether this is the case for all patients with severe influenza or if this was specific to these young previously healthy individuals.

A pandemic provides the opportunity to study a large number of patients. This larger group can then be separated into subsets of patients with mild and severe disease and those with and without risk factors. This allows us to identify defining immune features for our group of interest. In this study, performed during the second wave of the 2009 pandemic, an extensive range of cellular immune responses in freshly isolated blood cells was analyzed at the point of admission to hospital. These human findings were then further explored in murine models of mild and severe infection. Our studies show that a TNF-α^hi^ M1-like monocyte response is a key determinant of disease severity in the young, previously well adults with severe infection. Murine studies suggest that these cells contribute to a raised lung M1/M2 macrophage ratio and immunopathology of severe influenza.

## Results

### Monocytes and low-density granulocytes are significantly increased in previously healthy, young individuals with severe influenza.

**Figure 1:**
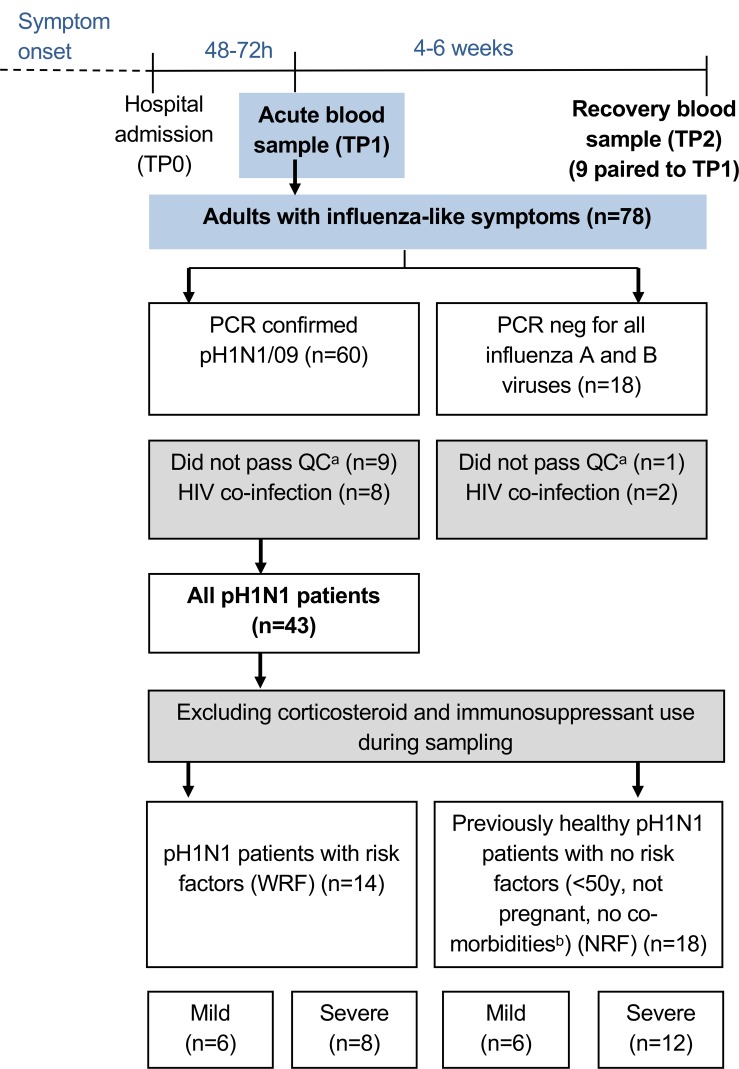
Flow chart of patient recruitment and grouping. All patients with suspected influenza A virus (IAV) infection (influenza-like symptoms) were sampled at TP1. ^a^Technical quality control (QC) required that all samples were processed without any freeze-thaw cycles, all baseline “fluorescence minus one” FACS controls were present, and control fluorochromes were at the expected intensity on the day of acquisition. ^b^Comorbidities in the study were hematological malignancies, asthma, chronic obstructive pulmonary disease (COPD), diabetes mellitus, cardiac failure, ischemic heart disease, and cancers. Patients with hypertension were not excluded. TP, time point; WRF, with risk factors; NRF, no risk factors.

The Mechanisms of Severe Acute Influenza Consortium (MOSAIC) was established to recruit patients with influenza-like illness hospitalized during the pandemic (2009–2010) and the postpandemic (2010–2011) periods of the pH1N1 outbreak (13, 14). Recruitment occurred in 11 London and Liverpool hospitals in the United Kingdom, from which fresh blood obtained within 72 hours of admission (time point 1 [TP1]) (Figure 1 and Table 1) was processed in one center (Oxford) for 78 cases. Samples from healthy noninfected controls were also sent in identical conditions. Disease severity was defined at the point of sampling according to criteria agreed upon by the MOSAIC consortium and based on the degree of respiratory compromise: grade 1, no respiratory compromise, i.e., normal oxygen saturation breathing room air; grade 2, respiratory compromise requiring supplemental oxygen with oxygen saturations ≤93% while breathing room air, with or without requirement for noninvasive mechanical ventilatory support; and grade 3, respiratory compromise requiring mechanical ventilatory support. We classified grade 1 as “mild” disease and grades 2 and 3 as “severe” disease.

All samples were analyzed fresh using flow cytometry for monocyte, neutrophil, NK, CD4, CD8, and CD8 effector T cell frequencies and IFN-γ ELIspot response to overlapping peptides of the entire pH1N1 proteome. Detailed definitions of immune populations are found in Table 2 and Supplemental Figure 1 (supplemental material available online with this article; https://doi.org/10.1172/jci.insight.91868DS1). Excess peripheral blood mononuclear cells (PBMCs) were cryopreserved for further use.

Of the 78 patients who presented with symptoms of influenza, 60 had pH1N1 virus infection confirmed by PCR. Of these, 8 were excluded because of HIV-1 coinfection and 9 did not pass quality control requirements for sample analyses (see Figure 1 legend for details), leaving 43 patients for our study.

We adopted two analytical approaches. First, we questioned which immune cells were significantly different between mild and severe patients in the whole cohort of IAV-infected patients (*n* = 43). Then, we excluded those who were on corticosteroids and immunosuppressants at the point of sampling, which could independently affect immune response, and divided the remaining patients into previously healthy individuals with no risk factors (NRF) (*n* = 18), referred to as the NRF group (Supplemental Table 1), and those with a variety of risk factors for severe disease, e.g., pregnancy, hematological cancers, or asthma (*n* = 14), referred to as the with risk factors (WRF) group (Figure 1 and Table 1). We noted but did not segregate patients by BMI (Table 1).

**Figure 2:**
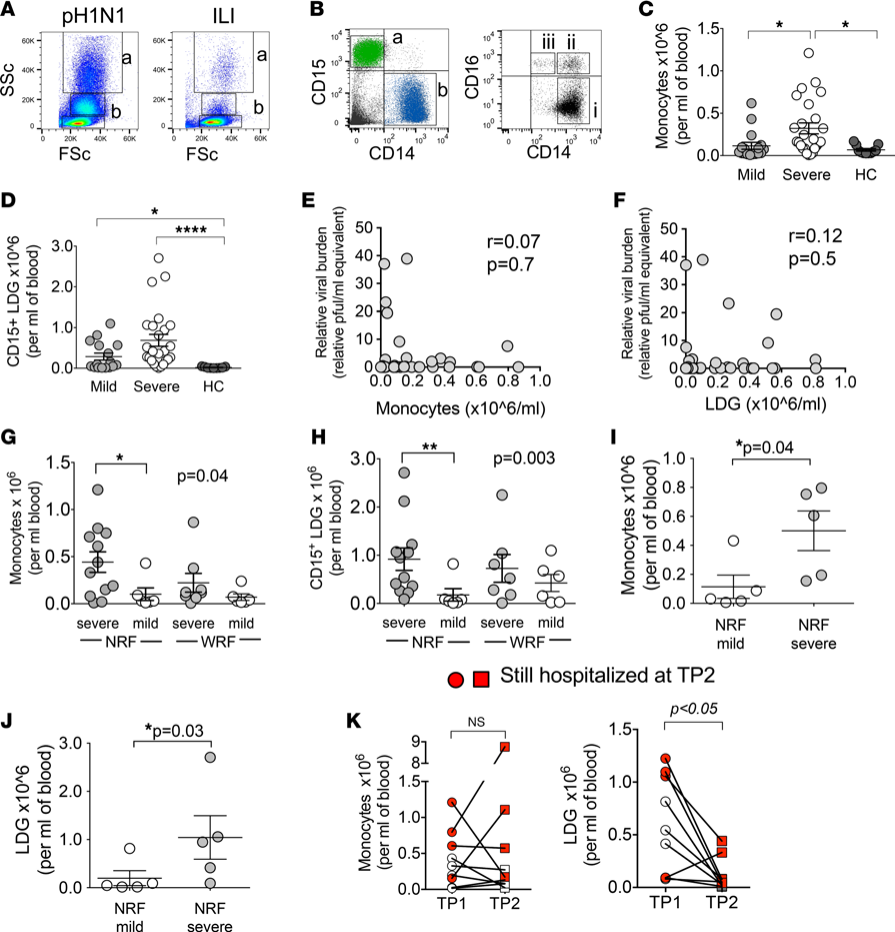
Sustained increase in monocyte levels in NRF patients with severe IAV infection. (**A**) Typical flow cytometry size-granularity plots from a severe pH1N1 and an ILI patient demonstrating excess of large, granular immune cell populations (gates “a” and “b”) for IAV-infected patients. ILI, influenza-like illness but PCR negative for influenza virus. (**B**) Gate “a” from **A** was identified as CD15^+^CD14^–^ low density granulocytes (LDG) and gate “b” comprised CD14^+^ (CD14^mid–hi^) monocytes. Gate “b” can further be divided into CD14^hi^CD16^–^ classical monocytes: “i”; CD14^hi^CD16^+^ intermediate or inflammatory monocytes, “ii”; and CD14^mid^CD16^+^ nonclassical or patrolling monocytes, “iii.” These gates were CD3^–^. (**C** and **D**) Circulating CD14^+^ (i.e., CD14^mid–hi^) monocytes and CD15^+^ LDG expressed as absolute numbers of cells per ml of blood for all pH1N1 patients (*n* = 43; *n* = 17 mild, *n* = 26 severe) and healthy controls (*n* = 12). (**E** and **F**) Relationship between viral load at TP0 (see Table 1) and circulating monocytes and LDG for 41 of 43 pH1N1 patients (viral load unavailable for 2 patients). Viral load relative to each patient was expressed as “relative PFU equivalents” (see Methods). (**G** and **H**) Number of circulating CD14^+^ monocytes and CD15^+^ LDG for NRF (no risk factors; *n* = 18) and WRF (with risk factors; *n* = 14) patients. (**I** and **J**) Monocyte and LDG numbers for *n* = 5 severe and *n* = 5 mild NRF patients normalized for time from the first symptoms. These patients were sampled between 8 and 18 days from the first symptoms. (**K**) Monocyte and LDG numbers at TP1 (admission) and 4–6 weeks later (TP2) for *n* = 9 patients from the NRF severe group. Filled symbols refer to patients still hospitalized at TP2. r values and significance were calculated using Spearman’s rank test. All values are mean ± SEM for normally distributed sets and median ± interquartile range for nonnormal distribution. *P* values were calculated using Kruskal-Wallis test and Dunn’s multiple comparison test (**C**, **D**, **G**, and **H**); Mann-Whitney (**I** and **J**); and Wilcoxon matched-pairs signed rank test for TP1 versus TP2 (**K**). *P < 0.05; **P < 0.01; ****P < 0.0001. TP, time point; HC, healthy control.

An overview analysis of the flow cytometry data revealed an excess of large, granular cells in PBMCs from many pH1N1-infected patients (Figure 2A). These cells can be classified as being of a monocytic (CD11b^+^CD14^mid–hi^CD15^–^) or neutrophilic (CD11b^+^CD14^–^CD15^+^) origin (Figure 2B). The large number of CD15^+^ neutrophilic cells was unexpected, since classical neutrophils do not colocalize with mononuclear cells following PBMC isolation by density gradient. These low-density granulocytes (LDGs) have also been identified by others in PBMCs from patients with severe sepsis, cancer, and HIV-1 infection (15–17).

Examining these and the other cellular immune responses in all patients with confirmed pH1N1 infection (*n* = 43), we found that monocytes and LDGs were the only significantly different immune populations between patients with severe and mild disease (Figure 2, C and D, and Table 2).

Viral burden (obtained from nasal swabs or nasopharyngeal aspirates 24 hours before TP1) was presented as “pfu/ml equivalents”, derived from a standard curve generated by seeding known amounts of pH1N1 RNA onto MDCK plaque assays.The severe group had a significantly higher viral load relative to the mild group, though no difference between NRF mild and NRF severe groups was seen (Supplemental Figure 2, A and B). Additionally, there was no relationship between viral load and when the samples were obtained (Supplemental Figures 2, C–F). Neither monocytes nor LDG levels correlated with viral load (Figure 2, E and F).

When we divided the patients into mild and severe NRF and WRF groups, excluding those on corticosteroids and immunosuppressants, the significant differences in monocyte and LDG levels between mild and severe cases were only found in the group of patients with NRF (Figure 2, G and H, and Supplemental Figure 3). This was also the case when monocytes and LDGs were analyzed as percentages of live cells in PBMCs (Supplemental Figure 4, A and B).

Because patients were hospitalized at different time points after the onset of symptoms, we questioned if the observed increase in monocyte and LDG levels was related to the length of time from infection. We found no correlation between monocyte or LDG levels and the length of time from onset of symptoms (r = 0.4; *P* = 0.11 and r = 0.3; *P* = 0.25, respectively, rank-sum correlation test). Importantly, when standardizing for time from first symptoms by examining a subset of patients sampled at similar points from first symptoms (*n* = 6 NRF mild patients [range: 8–18 days; average 11.6 days] and *n* = 5 NRF severe patients [range: 8–17 days; average 12.2 days]), we again observed a similar increase in numbers of monocytes and LDGs in severe NRF patients only (Figure 2, I and J).

Nine patients from the NRF group with severe disease consented to an additional blood sample 4–6 weeks after acute sampling (TP2), providing paired comparisons. There was no significant difference in monocyte levels between TP1 and TP2 (Figure 2K). Patients who were still hospitalized at TP2 had a significantly higher percentage of monocytes than those who had been discharged (Supplemental Figure 4C). In contrast, LDG levels were markedly decreased at TP2 (Figure 2K and Supplemental Figure 4D).

As more patients in the NRF group had increased BMI compared with the WRF group, we also questioned if BMI influenced the monocyte and LDG distribution in the group but did not find any significant association between these parameters or with severity (Supplemental Figure 4, E–I). Monocyte, but not LDG, levels remained significantly increased in NRF severe group when those with BMI >30 were excluded from analyses (Supplemental Figure 4, J and K)

Together, these results reveal that high levels of circulating monocytes and LDGs (independent of viral load) defined the immune profile of young previously healthy patients with severe disease. However, only high levels of monocytes were sustained in severe disease, while high levels of LDGs were specific to the earliest period of infection and returned to normal after this period (even in patients who remained ill).

### Monocytes from severe NRF patients show M1-like features.

**Figure 3:**
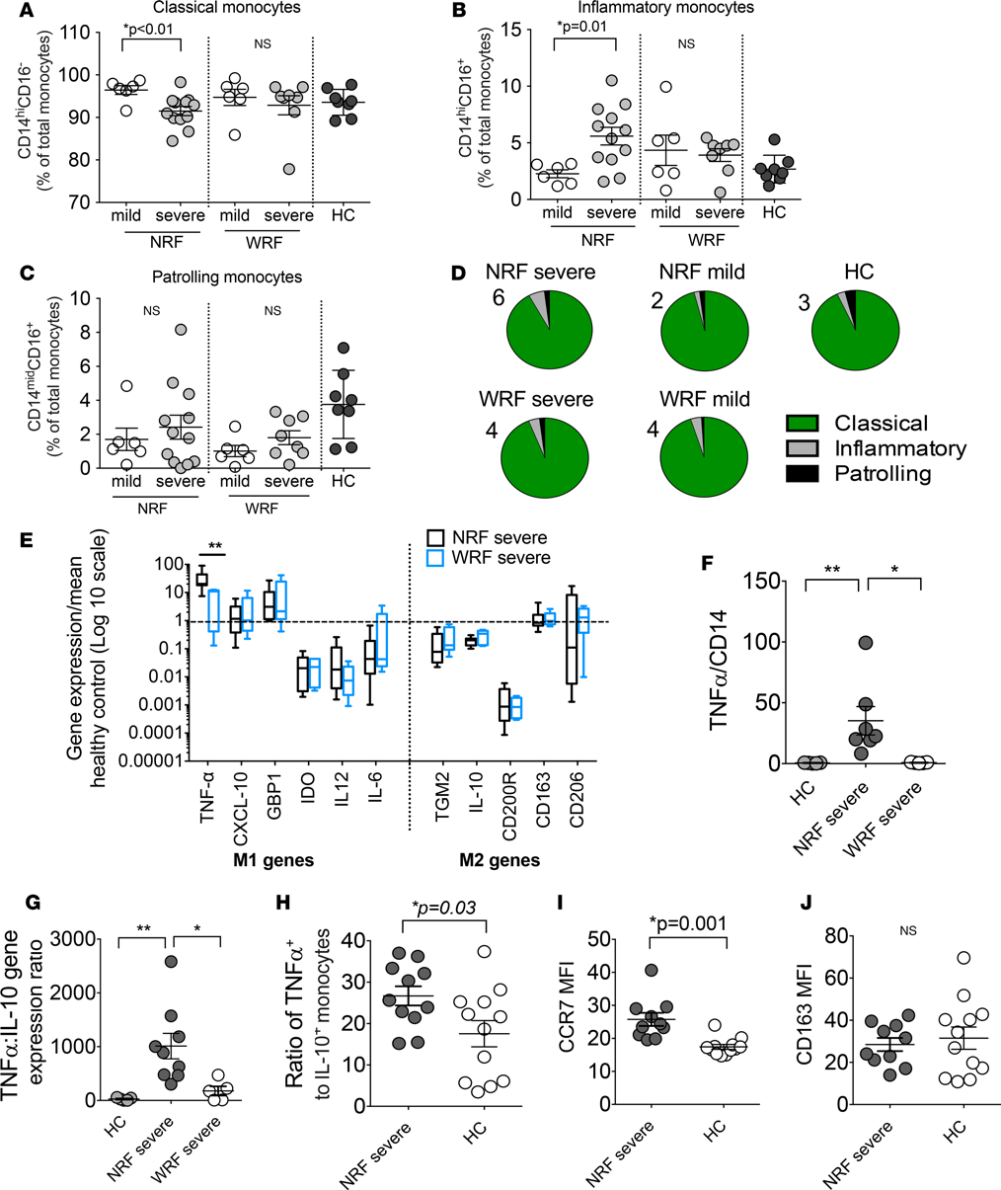
Monocytes in severe NRF patients are M1 like. (**A**–**C**) Classical, inflammatory, and patrolling monocytes in blood of mild and severe NRF and WRF patients. (**D**) Pie chart representation of classical, inflammatory, and patrolling monocyte frequencies in blood of NRF or WRF patients and healthy controls (HC) observed in **A–C**. Values refer to % of total monocytes. (**E**) Ex vivo expression of genes associated with M1 and M2 macrophage differentiation in CD14^+^ monocytes isolated from *n* = 10 NRF and *n* = 5 WRF patients with severe disease. Each gene is normalized to β-actin in the sample and then compared with the mean of the gene/β-actin of healthy controls (*n* = 9) (Mann-Whitney test with Bonferroni correction for multiple testing; ** adjusted *P* = 0.01). (**F**) TNF-α gene expression normalized to CD14 gene expression for each of the severe NRF and WRF patients and healthy controls. (**E** and **F**) Asterisks refer to statistically different genes comparing NRF severe and WRF sever. (**G**) TNF-α/IL-10 gene expression. (**H**) Ratio of TNF-α/IL-10 protein expression by intracellular cytokine staining. TNF-α and IL-10 expression (as cytokine-positive cells, as proportion of CD14^+^ monocytes) was measured following 6 hours of LPS stimulation of PBMCs. (**I** and **J**) Expression of M1 (CCR7 surface staining) and M2 (CD163 surface staining) markers on monocytes, measured by flow cytometry. *P* values were calculated using Kruskal-Wallis test and Dunn’s multiple comparison test for **F**–**G** and Student’s *t* test if data were normally distributed and Mann-Whitney test if not for **A**–**D** and **H**–**J**. *P < 0.05; **P < 0.01. NRF, no risk factors; WRF, with risk factors.

We next determined whether the increased numbers of monocytes from severe NRF patients corresponded to a particular phenotype. We first examined the frequencies of the well-characterized monocyte subsets: classical (CD14^hi^CD16^–^), intermediate or inflammatory (CD14^hi^CD16^+^), and nonclassical or patrolling (CD14^mid^CD16^+^) (18). There was a significant increase in the inflammatory subset in severe NRF patients, with a corresponding reduction in classical monocytes when compared with mild NRF and healthy controls (Figure 3, A–C). However, the inflammatory subset was small relative to the classical subset and made up less than 10% of monocytes for all groups (Figure 3D). We therefore hypothesized that there may be further differences, possibly within the classical subset, that were not captured by this division. Since macrophages can be classified as M1 or M2 according to specific surface markers secondary to IFN-γ/LPS or IL-4/IL-13 activation respectively (19), and since we have previously shown that some M1 and M2 features are conserved between circulating monocytes and tissue macrophages (20), we explored the use of M1 and M2 markers as a method of characterizing monocytes before they differentiate into macrophages. In the first step, we isolated CD14^+^ monocytes by positive MACS bead isolation from stored PBMCs of 10 severe NRF and 5 severe WRF patients (there were insufficient cells stored for the mild patients) and 9 of the healthy controls (all isolated and stored with identical methods) and examined ex vivo expression of 11 genes related to the M1 (*IL6*, *TNFA*, *IDO1*, *GBP1*, *CXCL10*, and *IL12B*) and the M2 (*TGM2*, *IL10*, *CD206*, *CD200R,* and *CD163*) macrophage subsets, selected from the recent macrophage activation guidelines (21).

Monocytes from severe NRF and severe WRF groups showed higher expression of some M1 (*TNFA*, *CXCL10*, and *GBP1*) genes compared with healthy controls (Figure 3E). *TNFA* was significantly increased in severe NRF compared with the severe WRF group. This was also the case when *TNFA* was normalized to *CD14* levels (Figure 3F). All but one of the M2 genes (*CD163*) were downregulated in the NRF severe patients compared with healthy controls (Figure 3E). However, the only significant difference between NRF severe and WRF severe groups was in *TNFA* expression. We then used *TNFA* and *IL10* as functional representatives for M1 and M2 phenotypes, respectively, and found that the ratio of *TNFA* to *IL10* expression was markedly increased in the severe NRF group compared with the severe WRF group (Figure 3G).

We used the remaining stored samples (*n* = 11 NRF severe patients; no WRF samples were available) to validate the *TNFA*/*IL10* gene expression ratio and to examine other representative surface protein levels for M1 (CCR7) and M2 (CD163) markers (21). The TNF-α/IL-10 protein ratio was increased in severe NRF patients compared with healthy controls by intracellular cytokine staining after 6 hours of LPS stimulation, due to both high TNF-α and a reduction in IL-10 levels in NRF severe patients (Figure 3H and Supplemental Figure 5, A and B). CCR7 expression on monocytes from severe NRF patients was also increased compared with healthy controls, but CD163 expression was not (Figure 3, I and J). As no samples were available from patients with mild disease (NRF or WRF), it was not possible to surmise that the enhanced M1/M2 ratio for severe NRF patients was not also found in mild NRF patients. However, even if this were the case, the absolute numbers of M1-like monocytes per ml in severe NRF is likely to be higher than that in mild NRF, given the significant difference in absolute monocyte numbers between these groups (Figure 2G). In 4 NRF severe patients, we had enough cells to test the difference between acute samples at TP1 and samples obtained 4–6 weeks later on TP2. For these patients, the TNF-α and IL-10 protein levels remained unchanged (Supplemental Figure 5, C and D), suggesting persistence, at least, of the TNF-α and IL-10 intracellular cytokine profile.

These findings show that monocytes in NRF patients with severe disease displayed significantly increased TNF-α and a net M1-like phenotype compared with uninfected healthy controls and severe WRF patients.

### Increased M1-like monocytes in blood of severe IAV infection in mice is accompanied by high M1 monocyte-derived macrophages in their lungs.

To strengthen the findings from the human studies and to specifically explore the relationship between M1-like monocyte and macrophage profile in the lungs, we turned to murine models of severe and mild influenza infection. This was particularly important, as we were ethically unable to sample the lungs of patients with severe influenza. We first questioned if M1-like monocytes were similarly raised in severe disease, and if this increase is associated with high M1 monocyte-derived macrophages (MDMs) in the lungs.

**Figure 4:**
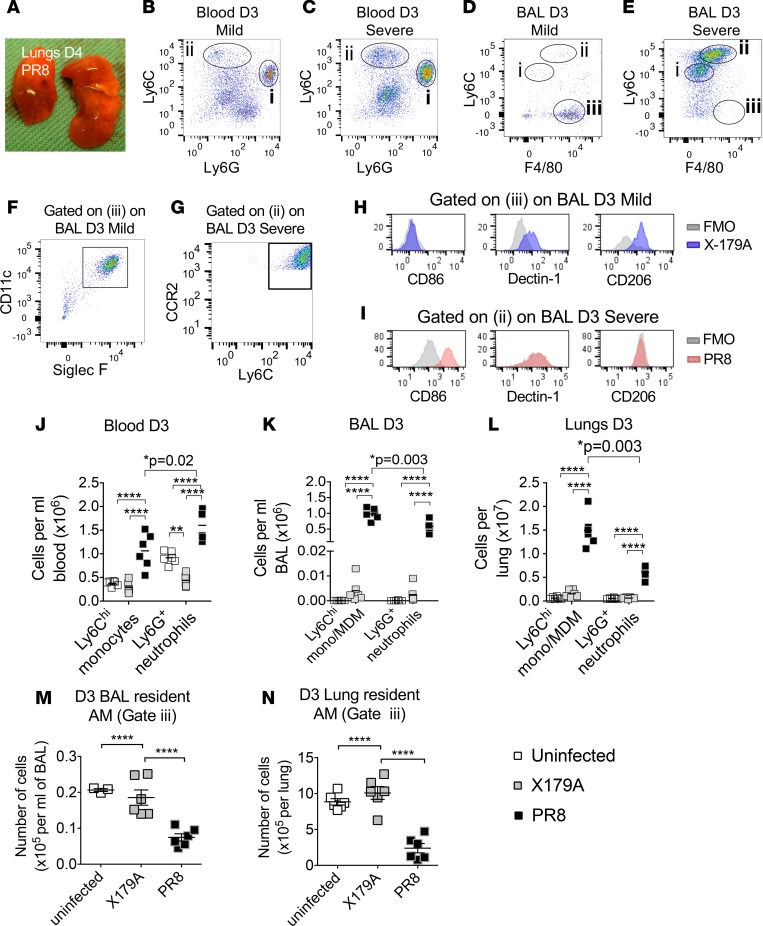
Increased circulating monocytes in severe IAV infection is matched by high levels of monocyte-derived macrophages in lungs but reduced resident alveolar macrophages. (**A**) Appearance of lungs from day 4 of severe IAV infection (PR8) showing hemorrhagic areas. (**B–G**) Gating of monocytes and neutrophils in blood of mild and severe murine models: classical Ly6G^+^ neutrophils (i) and CCR2^+^Ly6C^hi^Ly6G^–^ monocytes (ii) (equivalent to human CD14^hi^ monocytes) on FACS plot of red cell–lysed blood on day 3 of mild (X-179A) and severe (PR8) IAV infection. Alveolar resident macrophages are CD11c^+^SiglecF^+^ and monocytes or MDMs in BAL are CCR2^+^. Populations of cells in BAL on day 3 after infection: Ly6C^mid^F4/80^–^ neutrophils (i), Ly6C^hi^F4/80^mid^ differentiating monocytes/monocyte-derived macrophages (MDM) (ii), and Ly6C^–^ F4/80^hi^ resident alveolar macrophages (iii). Positive expression was defined against FMO samples to accommodate autofluorescence. (**H** and **I**). Alveolar macrophages (iii) express M2 markers, while monocytes/MDMs (ii) express M1 markers and are low in M2 expression. (**J–L**) Absolute numbers of monocytes and neutrophils in blood and monocytes/MDMs and neutrophils in BAL and lung digests on day 3 (D3) after infection with X-179A (mild) or PR8 (severe) and uninfected mice. Findings are from 2 experiments; *n* = 6 mice in total. Statistical comparison between monocytes and neutrophils in PR8 infection in **J**–**L** was performed separately and showed higher levels of monocytes/MDMs in BAL and lung digests compared with neutrophils (Mann-Whitney). (**M** and **N**) Number of Siglec F^+^ alveolar resident macrophages on day 3 in BAL and lung digests of mice. Statistical significance measured using 1-way ANOVA with Tukey test. Horizontal lines and error bars represent mean ± SEM for normally distributed sets and median ± interquartile range for nonnormal distribution. *P < 0.05; **P < 0.01; ****P < 0.0001. BAL, bronchoalveolar lavage; FMO, fluorescence minus one controls; AM, alveolar macrophages.

Immune cells in blood, bronchoalveolar lavage (BAL), and digests of unperfused lavaged lungs were examined in the early phase (day 3) of a well-established severe IAV infection model (A/PR/8/34 [PR8]) (Figure 4A and Supplemental Figure 6A) (22), compared with noninfected mice and those infected with a mild IAV (X-179A). X-179A–infected mice showed no change in weight loss and had significantly (*P* = 0.004) lower lung viral titers compared with PR8 infection but were able to protect against secondary challenge with PR8 (Supplemental Figure 6, B and C), indicating that an immune response was mounted during primary challenge. We defined the monocytes/MDM spectrum in blood, BAL, and lung digests as CCR2^+^Ly6C^hi^Ly6G^–^F4/80^–/mid^ cells, and the neutrophil and alveolar resident macrophages as Ly6G^+^Ly6C^hi^F4/80^–^ cells and Ly6C^–^F4/80^+^CD11c^+^Siglec F^+^ cells, respectively (23) (Figure 4, B–G). M1 macrophages were defined as CD86^+^Ly6C^hi^ cells and M2 macrophages as CD206^+^Ly6C^hi^ cells, based on established reports (24, 25).

As with others (26), we observed that Siglec F^+^ alveolar resident macrophages bore an M2-like phenotype with high expression of Dectin-1 and CD206 (Figure 4H), in contrast to infiltrating Ly6C^hi^ monocytes/MDMs, which were M1 like, with increased expression of CD86 but low Dectin-1 and CD206 expression (Figure 4I).

Similar to our findings in humans, monocytes and neutrophils in the blood were significantly increased in severe infection compared with mild infection (Figure 4J). This was accompanied by high levels of monocytes/MDMs and neutrophils in the BAL and lung digests (Figure 4, K–L); however, in the lungs, monocyte/MDM levels were in excess of those of neutrophils (Figure 4, K and L). In contrast to monocytes/MDMs, alveolar resident macrophages in severe disease were significantly reduced compared with mild infection on day 3 of infection (Figure 4, M and N).

**Figure 5:**
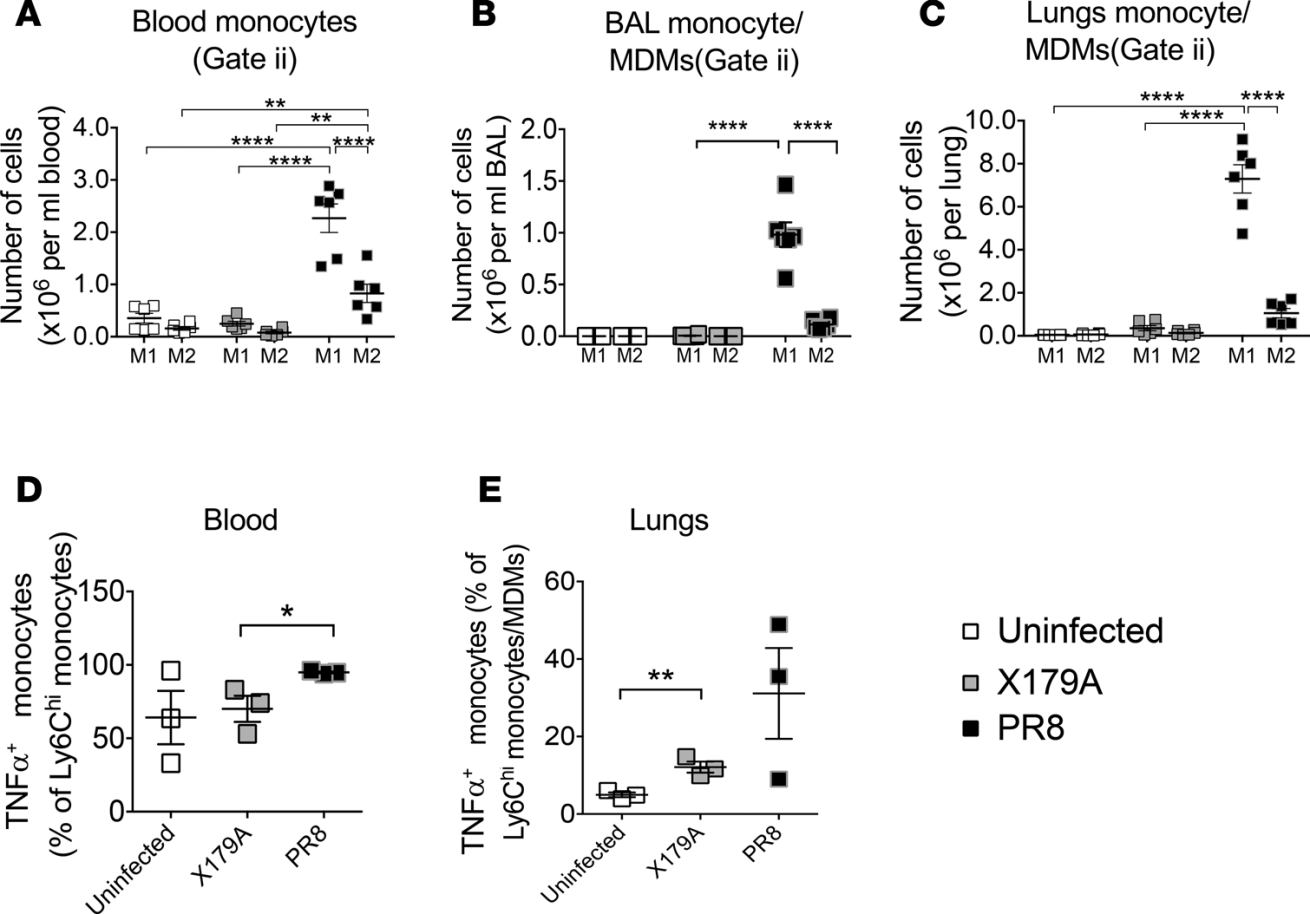
Blood monocytes and lung monocyte/macrophages are M1 like. (**A–C**) Number of M1 and M2 monocytes/MDMs on day 3 in blood, BAL, and lung digests. M1 monocytes were defined as CD86^+^Ly6C^hi^ cells and M2 monocytes as CD206^+^Ly6C^hi^ in appropriate gates from Figure 4, B–E. 2 experiments; *n* = 6 mice in total. (**D** and **E**) TNF-α production measured by flow cytometry intracellular cytokine staining after 6 hours of LPS in blood and ex vivo in lungs. Gated on Ly6C^hi^ monocytes from blood. Statistical significance measured using 1-way ANOVA with Tukey test. Horizontal lines and error bars in graphs represent mean ± SEM for normally distributed sets and median ± interquartile range for nonnormal distribution. *P < 0.05; **P < 0.01; ****P < 0.0001. BAL, bronchoalveolar lavage; MDM, monocyte-derived macrophage.

Similar to humans, monocytes and MDMs in blood of mice with severe disease showed a significantly higher M1/M2 phenotypic ratio (Figure 5A). This was matched by a high M1/M2 phenotypic ratio in the monocyte/MDM cells in BAL and lung digests of mice with severe infection compared with those with mild infection (Figure 5, B and C). LPS-induced TNF-α production by blood monocytes was significantly higher in mice with severe disease (Figure 5D). This was matched by increased ex vivo production of TNF-α from monocytes/macrophages in lungs of PR8-infected mice (Figure 5E) although this did not reach statistical significance.

These data show that, as in human disease, M1-like circulating monocytes were increased in the acute phase of severe disease in mice. This was matched by high M1 monocytes/MDMs in the lungs. Alveolar resident macrophages with an M2 phenotype were rapidly depleted in the acute period of severe but not mild infection, amplifying the M1/M2 macrophage ratio.

### Transfer of M2 macrophages to lungs during the acute phase of severe IAV infection reduces disease severity and improves outcome.

**Figure 6:**
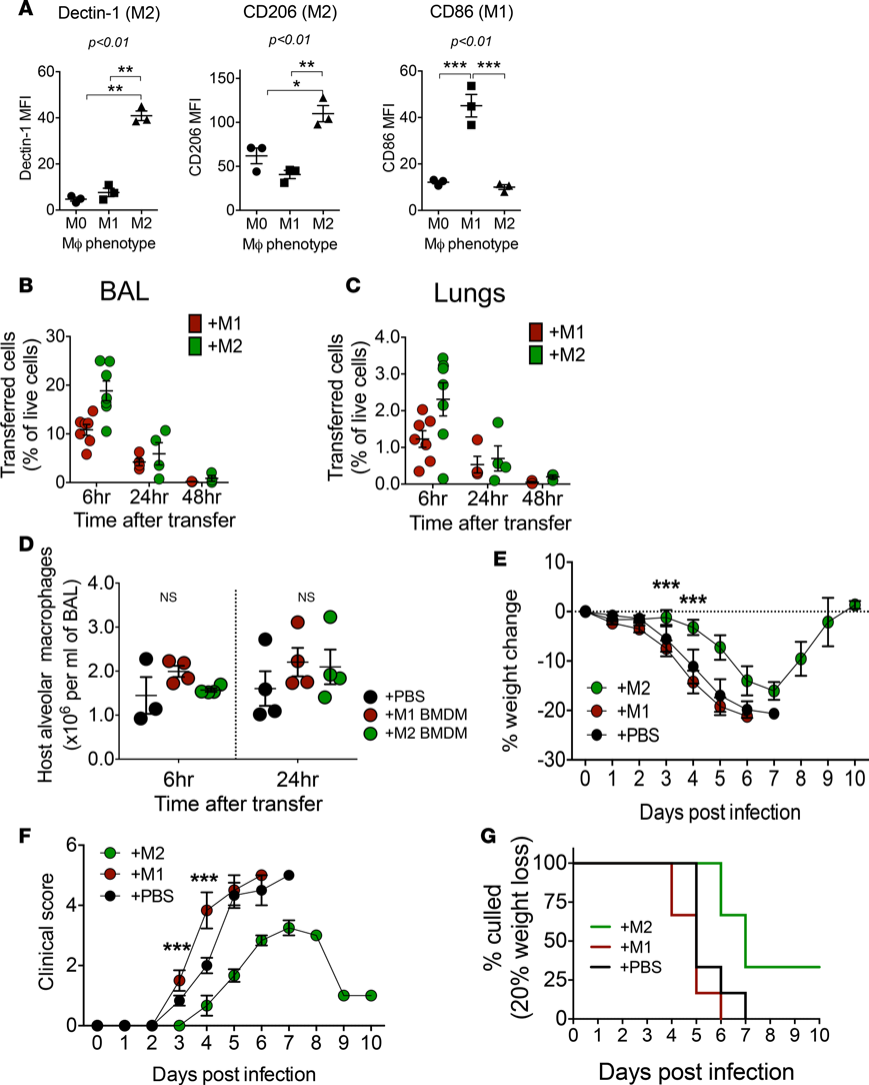
M2 macrophage transfer to lungs of infected mice improves disease outcome. (**A**) M1 and M2 markers on bone marrow–derived macrophages (MØ) used for adoptive transfer. Graphs show mean ± SEM from *n* = 3 preparations of bone marrow pooled from 4–6 mice per preparation. (**B** and **C**) Number of transferred cells (identifiable as CD45.1^+^ cells) in BAL and lung digests of mice 6 and 24 hours after administration of M1 BMDMs (+M1) or M2 BMDMs (+M2). *n* = 6 mice per group; 2 separate experiments. (Absolute values are given in Supplemental Figure 6, D and E.) (**D**) Numbers of resident alveolar macrophages in host mice after M1 BMDM or M2 BMDM transfer. 2 experiments, total of 4–6 mice at each time point for **B**–**D**. (**E–G**) Weight loss, clinical scores, and percentage of mice culled after adoptive transfer of M1, M2 BMDMs, or PBS. Clinical course was best in the M2-transferred group compared with PBS or M1 groups, with statistically significant findings on days 3 and 4. On day 4, mice that received M1 showed significantly worse clinical scores compared with those that received PBS and M2: clinical score 0, healthy; 1, calm but still exploring; 2, slow and exploring less; 3, hunched and shivery; 4, inactive; and 5, inactive even with handling. Total of *n* = 6 mice per group, 2 experiments. Differences among the 3 groups were analyzed using 2-way ANOVA with repeated measures. Comparisons were performed up to day 4 when all 3 groups still had equal numbers of mice. All *P* values for multiple comparisons were <0.001 except for clinical score on day 3, where the *P* value for M2 compared with M1 and PBS *P* < 0.05. For **A** and **D**, statistical significance was measured using 1-way ANOVA with Tukey test. *P < 0.05; **P < 0.01; ***P < 0.001. BMDM, bone marrow–derived macrophage.

We next questioned whether increasing M1 or M2 macrophages during the early periods of infection altered immunopathology. We generated M2 bone marrow–derived macrophages (BMDMs) from CD45.1^+^ C57BL/6 mice by culturing bone marrow cells in M-CSF for 6 days and then with IL-4 and IL-13 (M2) for the final 24 hours. As a comparator, M1 BMDMs were adoptively transferred in another group of mice. M1 BMDMs were generated by culturing bone marrow cells with IFNg rather than IL-4 and IL-13. BMDMs were >95% F4/80^+^. M1 and M2 BMDMs showed increased CD86 or CD206 and Dectin-1 expression, respectively (Figure 6A).

2 × 10^6^ M1 BMDMs, M2 BMDMs (suspended in PBS), or PBS alone were administered intranasally to PR8-infected CD45.2^+^ C57BL/6 mice 1 day after IAV inoculation. Successful BMDM transfer was shown by presence of CD45.1^+^ cells among CD45.2^+^ host cells, observed in BAL and digests of lavaged lungs at the sixth and twenty-fourth hour after transfer (Figure 6, B and C, and Supplemental Figure 6, D and E). M2 macrophage transfer was more successful than that with M1, with the transferred cells making up nearly 20% of live cells in BAL compared with approximately 10% for M1. This may be due to the higher apoptotic rate for IFN-γ–stimulated M1 BMDMs. Neither group of transferred BMDMs was detectable in the lungs at 48 hours after BMDM transfer (i.e., by day 3 of infection). M1 and M2 BMDM transfer did not alter the number of host macrophages in BAL, which, in the first 2 days, consisted of only Siglec F^+^ alveolar resident macrophages (Figure 6D). This meant that inadvertently, M2 BMDM transfer increased the M2/M1 ratio more than M1 BMDM transfer increased the M1/M2 ratio during the first few days of infection.

Mice that received M2 BMDMs showed significantly less weight loss following infection (Figure 6E) and better clinical scores over 10 days (Figure 6F) compared with infected mice that received PBS or M1 BMDMs. Mice that received M2 BMDMs were strikingly different clinically than those that received M1 BMDMs — they were able to resist restraint, while M1 mice were sluggish, shivery, and showed little resistance to handling. All mice that received M1 and PBS reached their 20% weight loss limit by days 6 and 7, respectively, and were culled, while 25% of mice that received M2 never reached the 20% weight loss limit (Figure 6G).

These findings show that increasing the M2 macrophage levels in the lungs during the acute phase of severe infection improved disease course, supporting a role for M2 macrophages in modulation of immunopathology in severe IAV infection.

### Lungs from mice with high-pathogenicity H5N1 infection show enrichment of monocyte-related genes and high M1/M2 gene ratio.

Finally, to strengthen our hypothesis that a high M1/M2 macrophage ratio is involved in lung immune pathology, we questioned if these findings are also observed in another model of severe influenza, that caused by a high-pathogenicity H5N1 IAV infection, which is associated with 60% fatality in humans (27). Three groups of mice (*n* = 6 per group in category 4 biolab facilities) were inoculated intranasally with the low-pathogenicity IAV H1N1 (X-179A) (at 1 × 10^5^ times the 50% egg infectious dose/ml [EID_50_/ml]) per mouse), severe H5N1 (A/Vietnam/1203/2004) (at 1 × 10^5^ EID_50_/ml per mouse), or PBS. As expected, H5N1-infected mice developed more severe disease, with higher lung viral loads compared with X-179A–infected mice, despite the same inoculating dose (Supplemental Figure 7, A–C). Mice were sacrificed at day 0 (before infection) and day 3 and 5 after infection, whole lungs (without flushing or lavaging the lungs) were harvested from each mouse, and RNA was extracted for gene expression profiling.

**Figure 7:**
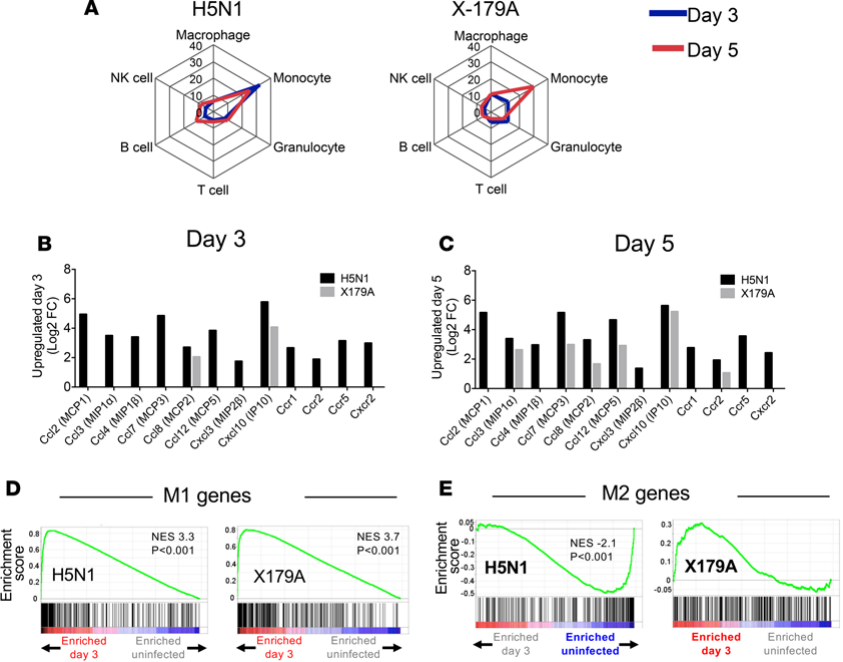
Lungs from high-pathogenicity H5N1 infection are enriched for monocytes and M1 genes, with downregulation of M2 genes. (**A**) Quantitative spider plot representation of the degree of enrichment of gene ontology (GO) terms for major immune cell subsets using the GOrilla online tool. Numbers represent enrichment scores. Granulocyte, regulation of granulocyte chemotaxis; Monocyte, regulation of monocyte chemotaxis; Macrophage, regulation of macrophage chemotaxis; B cell, regulation of B cell–mediated immunity; NK cell, regulation of NK cell–mediated immunity; T cell, positive regulation of α-β T cell activation. Days 3 and 5 refer to days after infection. (**B** and **C**) Expression of differentially regulated monocyte-attracting chemokines in lungs on days 3 and 5 after infection with H5N1 (black) or X179A (gray) relative to uninfected mice. (**D** and **E**) GSEA enrichment plots of M1 macrophage or M2 macrophage genes in lungs from H5N1- or X179A-infected mice on day 3, showing M1 enrichment of upregulated genes on day 3 for both H5N1 and X179A. This indicates an overrepresentation of M1 genes in both infections on day 3. In contrast, there was an underrepresentation of M2 genes in H5N1 but not X179A. This is one of 4 gene sets used to interrogate M1 and M2 gene enrichment (GSE51466) (all 4 are shown in Supplemental Figure 8). Enrichment score refers to the degree to which the gene set is overrepresented at the top or bottom of the ranked input list of genes. *n* = 3 mice per group for gene arrays. NES, normalized enrichment score (adjusted for gene set size or multiple hypothesis testing). Red bold or blue text indicates statistically significant enrichment of genes on day 3 after infection or in uninfected lungs, respectively.

We first examined the enrichment of immune cellular gene ontology (GO) terms in lungs using the Gene Ontology enRIchment anaLysis and visuaLizAtion (GOrilla) tool (28) and relevant GO terms (Supplemental Table 2). We found a marked polarization toward monocyte-related GO terms on day 3 of H5N1 infection, which remained skewed on day 5 (Figure 7A and Supplemental Table 2). In contrast, high polarization to monocytes only emerged on day 5 in X-179A infection. As the lungs were unperfused, these genes reflect expression in cells from blood within the lung vasculature, in addition to cells in the interstitium and alveolar space. This was complemented by upregulation of all chemokine genes involved in monocyte recruitment on day 3 in H5N1 infection, whereas only two genes (*CCL8* and *CXCL10*) were upregulated in X-179A infection at this time point (Figure 7B). By day 5, more of these genes were upregulated in mild disease but at lower levels than in H5N1 infection (Figure 7C).

Using gene set enrichment analysis (GSEA) (29), we then tested for enrichment of M1- or M2-specific genes in H5N1 or X-179A infection compared with uninfected lungs. Four gene sets encompassing the different methods of M1 and M2 activation were tested (Supplemental Table 3). Lungs from H5N1 and X-179A infection showed enrichment for genes associated with M1 macrophages on day 3 and 5 (Figure 7, D, and Supplemental Figure 8, A and B). However, H5N1-infected (but not X-179A–infected) mice showed significantly less involvement of M2 genes when compared with uninfected mice (Figure 7, E), and Supplemental Figure 8, A and B) consistent with the loss of M2-biased alveolar resident cells early in infection in the PR8 severe model (Figure 4, E, M, and N).

These findings show that, in comparison to other immune subsets, high and rapid expression of monocyte genes (possibly from cells carried by blood supplying the lungs), rather than neutrophils, and a high M1/M2 macrophage gene ratio in the lung, amplified by downregulation of M2 genes, were distinctive features of high-pathogenicity H5N1 virus infection. This provides further support for the link between high levels of circulating monocytes (relative to neutrophils) and high M1/M2 ratio during acute phase of infection, with disease severity in IAV infection.

## Discussion

Many of those who die from influenza are debilitated by co-existing medical conditions, old age, are very young, pregnant or suffering from some other known predisposition. It is highly unusual for previously healthy individuals to suffer life-threatening influenza disease, but these patients form a distinct and under-researched group. Of the 60 cases of severe influenza that we now report, 18 (30%) were without known risk factors (NRF) and were most severely ill — all required mechanical ventilatory support. Although it is thought that this group of severely affected, previously healthy adults is unique to pandemics, our study shows that, at least in the first postpandemic season, there was also a sizeable number of these patients. Middle-aged, previously healthy patients are also more highly represented in novel infections like the H5N1 and H7N9 outbreaks (30); although there are other factors at play in these outbreaks, for example, the high pathogenicity of the virus. We took advantage of the opportunity to study patients in the second wave of a low-pathogenicity IAV (pH1N1/09) pandemic, which made it less likely that severe infection was caused by the novelty or the inherent pathogenicity of the IAV strain. The study also benefitted from having enough patients to stratify into groups with and without risk factors and, within these, those with mild and severe influenza, allowing us to determine the immune profile unique of those severe patients with NRF. We show that the ability to mount a particular type of monocytic response (M1 like, TNF-α^hi^) is a key feature in severe IAV, which appear specific to these young previously healthy patients with severe disease. We note that there was a trend toward higher levels of T cells recognizing pH1N1 HA, NA and NP in PBMC (Supplemental Figure 3, D–I). This did not correlate with viral load but rather with the ratio of monocytes to T cells (Supplemental Figure 3J), so we speculate that presence of higher levels of monocytes may provide better antigen-presenting capacity in the assays. In fact, when effector CD8 T cells were examined, no differences were found between groups (Supplemental Figure 3C).

We also observed more females in the milder group (mild NRF and mild WRF) compared with the severe groups (Table 1) in our study. Gender differences in incidence, morbidity, and mortality have been discussed in a WHO report (31), which concluded that, on balance, morbidity is worse in childhood and old age for men and worse in middle age for women, possibly due to inclusion of pregnant women in epidemiology studies. In our NRF group, pregnancy was excluded, so the cohort supports the possibility that, in the NRF groups, severe disease occurred more commonly in male individuals. However, the small numbers preclude a robust interpretation.

The murine studies were important to strengthen our human findings, since we were constrained by the number of patients we had within one season and within the consortium. A pathogenic role for tissue macrophages per se in severe IAV infection is already shown by other studies (32–36), and this was not the intention of our murine studies. Rather, we questioned whether the high levels of circulating monocytes found in severe human disease with all the incumbent limitations of human studies (e.g., variation in days from infection) could be replicated in the cleaner murine studies. We also wanted to determine if these monocytes were similarly biased toward an M1-like phenotype (since this has not been shown before in circulating monocytes) and whether this is matched by high M1 MDMs in the lungs. We can conclude that high ratio of M1/M2-like monocytes is a feature of severe influenza infection and this is associated with high M1-like macrophages in the lungs.

The lung studies raised the potential significance of another finding in causation of severe disease — that of a near depletion of the M2-biased Siglec F^+^ alveolar resident macrophages in the early part of the severe but not mild infection. This loss has been reported before, although not in the context of its contribution as a M2 macrophage presence and not in direct comparison to milder models with lower viral loads (37). The murine studies did not allow for the kinetics of these cells to be observed, so alveolar resident macrophages may also eventually be depleted in the mild model. Although the frequency of Siglec F^+^ alveolar resident macrophages is low (Figure 4, M and N), their presence may modulate the immunopathology of infection by way of having M2-like antiinflammatory properties (23). This study does not prove that the Siglec F^+^ macrophages are involved in improving outcome, merely that presence of M2 BMDMs for a brief point (in fact, just before the depletion of M2-biased resident alveolar macrophages occurred) improved disease course. Soluble factors produced by these cells, possibly IL-10 (as observed by others) (38), may have an influence on the immune profile beyond this brief period. Another possibility is that the transferred M2 macrophages increased phagocytosis of virus-infected cells. We note that the M1 MDM transfer did not result in a significantly worse outcome, although the trend of clinical scores and weight loss was toward worse disease. One explanation could be the lower numbers of transferred M1 MDMs (Supplemental Figure 6, D and E), possibly due to the higher apoptotic rate of these cells (39). Another possibility is the relatively low net increase in M1 macrophages provided by the transferred cells within a mouse system that already had very high levels of M1 macrophages. Thus, although there was a clear association between high M1-like monocytes in the blood and high M1 macrophages in the lungs and disease severity, the limitations of the M1 macrophage transfer meant that the murine study falls short of showing that M1 macrophages per se worsens disease.

We used the “M1” and “M2” nomenclature in our study but acknowledge that there are clear overlaps in phenotypes and many functional subtypes that are not covered by this categorization (40). However, they remain the most established method of categorization, and we use it to provide a guide to the functional polarization of macrophages in vivo where M1 are proinflammatory and M2 prorepair or antiinflammatory. Large sets of genes define these subtypes, and our choice of surface phenotypic markers reflects accepted representative markers for these subtypes (21, 24, 25). In mice, M2 surface markers are established, with CD206, Dectin-1, and CD163 being used together or singly in most publications in this area. M1 is less easy, particularly for representative surface markers rather than gene expression. We chose CD86 (as did others, refs. 24 and 25) because of a well-designed study by Kigerl and colleagues (24) showing infiltration of CD86^+^ (M1) tissue macrophages in vivo, along with gene expression profiling of all M1 and M2 genes from landmark studies on M1 and M2 macrophages (40).

Our findings provide a cellular pathway for the so-called immune storm in these severely ill patients (12) and pinpoint a pathway that could potentially be targeted for immune-modulatory therapy. We note that both monocytes and LDGs were increased. However, we focused on the monocytes, as their numbers were still increased 4–6 weeks after acute infection (in patients who were still in hospital) (Figure 2K), providing a stronger correlate to severity. The identity of the LDGs is also unclear; potentially, they are a mixture of myeloid-derived suppressor cells (15, 41), immature neutrophils (17), activated neutrophils (42), and neutrophils undergoing NETosis, which have a lower density than neutrophils (43). Our murine studies subsequently showed that monocytes were more numerous than neutrophils in the lungs of severe IAV infection (severe PR8 infection, as well as severe H5N1 infection.

In humans, no relationship was found between a high viral load and monocyte response. Our viral load values refer to the viral burden at the point of admission and sampling for the cellular studies. These values are accurate to examince the relationship between viral burden (relative to other patients within the cohort sampling) and immune parameters, which were measured at the same time. They are likely but may not relate to, the initial viral load at the point of infection. Within these limitations, the lack of relationship between viral load and monocytic response suggest that host predisposition to a high monocytic response could be at play. This could be due to host genetic variants that increase the sensitivity of monocytes to chemoattractants, such as CCL2 (44), or, as shown in our recent studies, polymorphisms in IFITM3, a virus restriction factor, which was associated with increased CCL2 levels, increased viral load, high levels of monocytes, and severe disease (13, 45). Recently, polymorphisms in the TNF-α gene were found to be overrepresented in severe disease in a Mexican 2009 pandemic cohort (which comprised mainly patients with no comorbidities) (46) and could be a cause of the extremely high expression of TNF-α in monocytes derived from severe NRF patients in our study. Direct contribution of TNF-α^hi^ monocytes to disease severity is strengthened by our murine findings, in which circulating monocytes uniformly secreted more TNF-α protein upon ex vivo stimulation. This also resonates with findings from Aldridge et al., in which severe lung injury was observed in conjunction with high levels of TNF-α/iNOS-producing DCs (47).

A role for monocytes in causation of immune pathology in young previous healthy individuals is also supported by a recent community-based study utilizing a broader age range (4 weeks to 71 years). Here, Oshansky and colleagues showed that young age and nasal monocyte response correlated most strongly with disease severity (48). Infants and young children mounted a high innate immune response, with high levels of monocytes in nasal lavage that were unrelated to viral load. Together with our findings specifically in young previously healthy adults, the data suggest that a relatively inexperienced adaptive immune system may be a stage for a larger, persistent innate immune response and that monocytes are the chief players.

Increased monocyte levels are an eminently modifiable feature in humans, and ultimately this study concerns the identification of such a pathway. CSF-1 receptor inhibitors (already in clinical trials) (49) can reduce differentiation to macrophages, and their use can be guided by monocyte levels, which can act as a biomarker and indicator for treatment. Nebulized corticosteroids used for many years by clinicians for asthmatics could also potentially be an M2 macrophage-inducing agent and be delivered specifically to the lungs. Our M2 BMDM transfer findings suggest only a small amount of M2 macrophage in the lungs could change disease course. Adoptive transfer of M2 macrophages, or reprogramming of M1 macrophages toward M2, has successfully alleviated disease in other M1-biased conditions, such as obesity (50), RSV (51), and atherosclerosis (52). While multiple pathways are likely to be ultimately responsible for influenza pathogenesis, our study suggests that targeting macrophage reprogramming could be a useful therapeutic intervention during severe disease. While associated with pathogenesis, TNF-α neutralization has not been successful in influenza, and, in fact, targeting a single inflammatory mediator has often been met with little success (53). By targeting the macrophage polarization spectrum, multiple pathways could be altered with potentially greater and more specific effect.

Finally, we note that TNF-α was more highly expressed in circulating monocytes from the severe NRF group compared with the WRF group, highlighting TNF-α^hi^ monocytes as a potential specific biomarker for this group. This is significant, as currently there are no predictors for life-threatening disease in otherwise healthy people with influenza.

In conclusion, our studies identify increased M1-like monocytes as an important correlate of disease severity in young previously healthy adult patients with life-threatening disease and suggest that this increase leads to raised M1/M2 macrophage ratio in the lungs and contributes to disease severity in these patients. This provides a possible mechanistic cause for severity in these patients, a potential early identifier and a modifiable immune pathway for therapeutic targeting.

## Methods

### Human IAV study design.

Patients with pandemic influenza A/CA/07/09 (pH1N1) infection who required hospitalization in England between November 2010 and February 2011 were recruited via MOSAIC. Human sample size was not predetermined, as the study was designed to capture the largest possible population of a rare subset of patients (previously healthy patients who required hospitalization) during the second wave of the influenza pandemic. All blood samples (including healthy controls) were collected in sodium heparin tubes, placed in a BioJar with cool gel packs, and transported by courier immediately to Oxford, where they were processed within 4 to 6 hours of venesection. All samples from MOSAIC were tested for pH1N1 by PCR.

### Determination of viral loads in humans.

Molecular viral presence was determined from nasal swabs or nasopharyngeal aspirate obtained at hospital admission 24–48 hours before TP1 using diagnostic qRT-PCR performed at West of Scotland Specialist Virology Centre. Viral nucleic acid was extracted using the Qiagen MDx Biorobot automated extractor with the QIAamp Virus MDx Kit according to the manufacturer’s instructions. qRT-PCR reactions were set up to a total volume of 15 μl using the Qiagen One-Step RT-PCR kit, using primers (influenza A matrix [M] or pH1N1 neuraminidase [NA], ref. 24) as described previously (54) on an ABI Prism 7500 SDS real time platform (Applied Biosystems). Positive samples were confirmed by detection of influenza A M or NA gene or rising serology titers where available. For viral load quantitation, we first derived the crossing threshold (Ct) value (at the inflexion spot of the sigmoid amplification curve to capture the point at which DNA amplification is exponential) performed in a batched assay as a relative expression of viral burden against each sample. This was then measured against a standard curve of Ct value to PFU/ml generated by measuring PFU when known amount of pH1N1 was inoculated in MDCK cells. The results are presented as “relative PFU equivalents,” the values being relative to each other in the batched assay.

### IFN-γ ELISPOT assay.

Following PBMC extraction, ex vivo ELISpot assays were performed on fresh cells using methods described previously (55). Plates were coated the day before with IFN-γ capture antibody (1-D1K; MabTech) at 10 μg/ml and blocked with RPMI/10% FCS. 2 × 10^5^ PBMCs were added in duplicate to each well with peptides at a final concentration of 2 μg/ml. Plates were incubated for 18 to 20 hours at 37°C. Culture media was used as a negative control, and 1 μg/ml PHA was used as a positive control. Results were expressed as spot-forming units per 10^6^ PBMCs after background subtraction. For stimulation with pH1N1-specific peptides, sequences from the full A/CA/04/2009 proteome were split into overlapping peptides of 18–20 mers and divided into pools comprising HA, NA, NP, NS, PA, PB, and M. Only results derived from assays that passed the standard quality control of an appropriate positive or negative control response and background that did not reduce clarity of spot-forming units were used.

### Flow cytometry.

PBMCs were extracted using density gradient separation for same day flow cytometry phenotyping. For surface antigen staining, cells were incubated for 20 minutes at 4°C in the dark with fluorochrome-conjugated mAbs in 96-well round-bottom plates in FACS buffer (PBS containing 10% FCS and 1% BSA). For detection of intracellular cytokines, cells were fixed following surface staining in 1% formaldehyde (Sigma-Aldrich) for 15 minutes at room temperature, washed, and incubated for 30 minutes at 4°C in FACS buffer containing 0.5% Saponin (Sigma-Aldrich) and fluorochrome-conjugated mAbs. Intracellular cytokine staining was performed on cells following a 6-hour incubation with or without 1 μg/ml LPS in both human and mouse studies. In human studies, 10 μg/ml Brefeldin A was added for the last 4 hours of culture. For TNF-α detection from mouse blood and lungs, Brefeldin A was included in all buffers used in sample in all buffers used in sample collection, processing and subsequent ex vivo stimulation.

In murine flow cytometry experiments, nonspecific antibody binding was corrected for using FC block (anti-CD16/32, eBiosciences). In both human and mouse studies, fluorescence minus one controls were used to identify positive populations and account for autofluorescence. Samples were acquired on a CyAn analyzer (Beckman Coulter) or a 4-laser configuration LSR-II (BD Biosciences) and analyzed using Flowjo V10 (Tree Star). In all instances, data analysis were performed on single live cells by gating out doublets and dead cells. All freshly acquired samples had greater than 98% viability.

Antibodies used are listed in Supplemental Table 4 (human) and Supplemental Table 5 (mouse).

### qPCR.

RNA was extracted from purified CD14^+^ MACS-isolated monocytes from defrosted PBMCs or from homogenized lung tissue from mice using TRIzol reagent (Ambion) and reverse transcribed to cDNA using the SuperScript III First-Strand Synthesis System (ThermoFisher Scientific), following the manufacturer’s instructions. qPCR assays were set up using SYBR Green master mix (ThermoFisher Scientific) and analyzed on the 7900HT Fast Real-Time PCR System with 384-well block module (human CD14^+^ monocytes) or the 7500 Fast Real-Time PCR System (mouse lungs). Primer sequences for human monocytes are listed in Supplemental Table 6. Fold changes were analyzed using the 2^–ΔΔCt^ method. Primers for influenza matrix gene in mice have been described previously (56).

### Murine IAV models.

Seven to eight-week-old female C57BL/6 (CD45.2) mice and B6SJL (CD45.1) mice were obtained from breeding pairs originally purchased from The Jackson Laboratory. Mice were randomly allocated to infection or control groups. Investigators were not blinded to group allocation but were unaware of outcome (mild, severe, uninfected) during processing of tissues and analysis by flow cytometry. All experiments were designed in keeping with the NC3R guidelines. IAV was administered to mice intranasally in a total volume of 40 μl as previously described (22). Influenza A (H1N1) X-179A, a A/California/07/2009 — A/Puerto Rico/8/34 reassortant, and influenza A (H5N1) A/Vietnam/1203/2004 were all used at 1 × 10^5^ EID_50_/ml. Influenza A (H1N1) A/Puerto Rico/8/34 (PR8) was used at 1 × 10^5.35^ EID_50_/ml (severe model) or 1 × 10^3.25^ EID_50_/ml (macrophage transfer model) in the adoptive transfer experiments to reduce morbidity and cull rate for the model. PBS was used to inoculate “uninfected” mice. BAL was collected as previously described (22). Blood was collected in EDTA following cardiac puncture, and lungs were cut into 1-mm^2^ pieces and digested in Collagenase A (Roche) (1 mg/ml in PBS) and DNAse I (Sigma-Aldrich) (200 μg/lung) at 37°C.

### Murine macrophage generation and adoptive transfer.

Macrophages were generated from bone marrow isolated from femurs and tibias of 7-week-old female B6SJL (CD45.1) mice by culturing in M-CSF (40 ng/ml) for 6 days in 6-well low-adherence plates (Corning). After this, macrophages were differentiated over 24 hours in IFN-γ (10 ng/ml) for M1, IL-4 and IL-13 (10 ng/ml each) for M2, or nothing for M0. 2 × 10^6^ differentiated macrophages were then transferred intranasally to C57BL/6 (CD45.2) mice 1 day after PR8 infection.

### Murine lung tissue microarray.

Lungs were removed from euthanized mice, cut into 1-mm^2^ pieces and placed in RNAlater for RNA extraction using TRIzol reagent (Ambion). Lungs were homogenized using glass homogenizers and passed through a QiaShredder (Qiagen) for complete tissue disruption. 200 μl Chloroform was added to each sample to isolate the aqueous phase containing RNA, which was transferred to a new tube on ice. Isopropanol was added to precipitate RNA, and the RNA pellet was washed in 75% ethanol before resuspension in Nuclease-free water for quantification and quality assessment using Nanodrop and Agilent 2100 Bioanalyzer instruments. Intact nondegraded RNA (RIN >8) was used. For microarray, GeneChip whole-transcriptome (WT) target preparation was performed using the WT PLUS Reagent Kit (Affymetrix) following the manufacturer’s instructions, using 100 ng input RNA. Fragmented, labeled samples were hybridized onto Mouse Gene 1.0 ST Array chips, stained, and washed using the GeneChip Fluidics Station 450 and the fluorescent signals were captured as data image files for analysis with the Affymetrix Gene Expression Console.

For H5N1 lungs, RNA samples from each group were pooled to comply with low handling protocol in category 4 laboratories and hybridized onto one cartridge. For X-179A lungs, each biological replicate was hybridized separately (*n* = 3). Both sets were compared with their own uninfected controls.

### Microarray analysis.

Microarray data were analyzed with Affymetrix Expression Console Software and R program (https://www.r-project.org/) to identify differentially expressed genes. Ranked gene lists were created between day 3 or day 5 H5N1 or X-179A lung samples and lungs from uninfected mice for GSEA (29) and GOrilla (28) analysis. For GOrilla analysis, GO terms related to monocytes, macrophages, B cells, T cells, NK cells, and granulocytes were examined. GSEA was performed using open source software (v5.0, Broad Institute, http://www.broadinstitute.org/gsea/index.jsp). Ranked gene lists were computed with genes highly expressed in M1 or M2 macrophages, identified from published data sets (NCBI’s Gene Expression Omnibus accession GSE35495, GSE32690, GSE63245, and GSE51466), which were analyzed with GE02R. For this, M1 macrophages were compared with M2 macrophages to identify genes exclusively upregulated by either subset. Genes at adjusted *P* < 0.05 and logFC >1.5 were used for analysis. Where the number of genes was above 500, the top 500 genes were used for input into GSEA. Lists of genes are included in Supplemental Table 7. GOrilla analysis was performed online (http://cbl-gorilla.cs.technion.ac.il/) to identify significantly enriched GO terms, which are manually searched for terms relating to “monocyte,” “macrophage,” “neutrophil,” “granulocyte,” “T cell,” “B cell,” or “NK cell.” Heatmaps were created using Mev (http://mev.tm4.org/).

The data set and technical information according to the Minimum Information about a Microarray Experiment requirements are available at the Gene Expression Omnibus website (https://www.ncbi.nlm.nih.gov/geo/; accession GSE70882).

### Statistics.

All statistical analysis was performed using GraphPad Prism 5.01 (GraphPad) or R programming. Normality of data distribution was first tested using the D’Agostino and Pearson omnibus normality test, and equality of variance was examined using F test. Normally distributed data sets were compared using 2-tailed Student’s *t* test when comparing two groups or 1-way or 2-way ANOVA when comparing multiple groups. Where comparisons included data not normally distributed, the Mann-Whitney test was used to compare data sets. Where multiple data sets included data that were not normally distributed, Kruskal-Wallis with Dunn’s multiple comparison post-test analysis was performed. All tests performed were 2 sided. Corrections for multiple comparisons in large data sets (Supplemental Table 2 and the gene expression data) are specified in legends for the data shown. A *P* value of less than 0.05 was considered significant. Error bars on graphs represent SEM unless otherwise stated. During data analysis, investigators were unaware of outcome groups (mild, severe, healthy) for flow cytometry and ELISPOT studies until analysis was complete.

### Study approval.

The MOSAIC study was approved by the NHS National Research Ethics Service, Outer West London Research Ethics Committee, UK (09/H0709/52, 09/MRE00/67). All adult subjects provided informed consent, and a parent or guardian of any child participant provided informed consent on their behalf. Informed consent was given in written form in all cases. Where the patient was incapacitated, “assent” from relatives, friends, or welfare attorneys was obtained in written form. This was approved by the Oxfordshire Research Ethics Committee, UK.

H5N1 mouse experiments were performed under substantial severity limit protocols approved by the Home Office under the auspices of the UK Animal Scientific Procedures Act 1986 (PPL 30/2407) in specific containment facilities at the National Institute of Medical Research, Mill Hill, under Specified Animal Pathogen Orders license PATH/57/2009/1 at biosafety level 4. For all other murine experiments, mice were maintained at the Biomedical Services Unit at the John Radcliffe Hospital (Oxford, UK) and were used according to established University of Oxford institutional guidelines under the authority of a UK Home Office project license. The Home Office project license for all experiments allowed up to 20% weight loss. Specific permission was given for H5N1 experiments where 35% weight loss was allowed.

## Author contributions

SLC designed and performed experiments, analyzed data, and wrote and revised the manuscript. JD organized and obtained human samples and clinical data. WLK performed H5N1 experiments. KHB and AB performed experiments and analyzed data. ER performed the overall statistical analyses. FOM provided reagents and intellectual input. LD led the organization of clinical demographics of human samples. TJP performed ELISPOT experiments and analyzed the ELISPOT output. MB and JM provided reagents and contributed to the design of the category 4 H5N1 experiments. RE and WB led the viral load studies with input from CT and MZ. TD provided intellectual input. FYL provided intellectual input and edited the manuscript. ST contributed to microarray analysis. VC provided intellectual input. PJO led the MOSAIC consortium and edited the manuscript. AJM and LPH led the Cellular Immunology work package in MOSAIC. LPH conceived the idea for this paper; supervised the work; led study design, analysis, and interpretation; and wrote and revised the manuscript.

## Supplementary Material

Supplemental data

Supplemental Table 7

## Figures and Tables

**Table 2:**
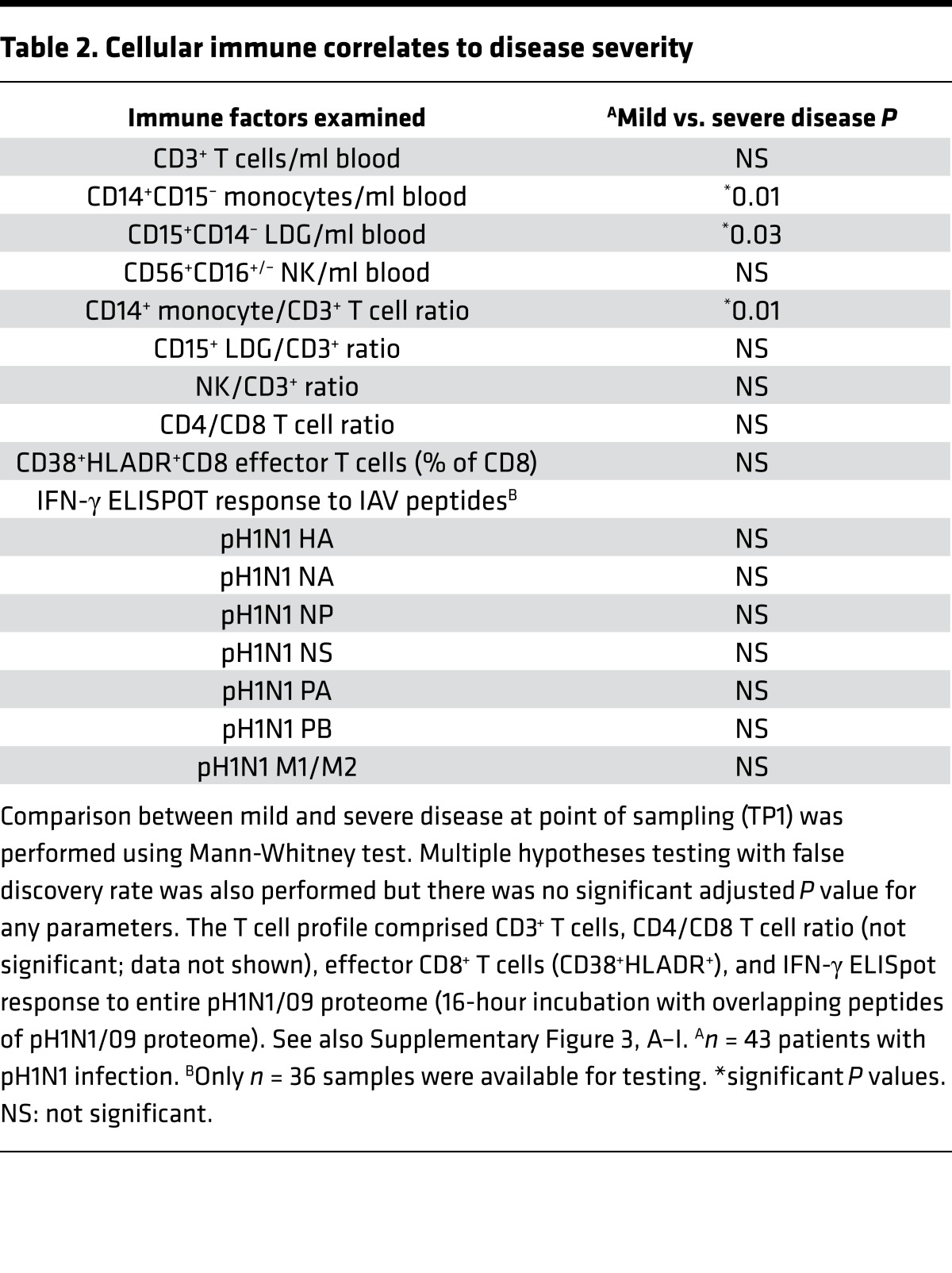
Cellular immune correlates to disease severity

**Table 1:**
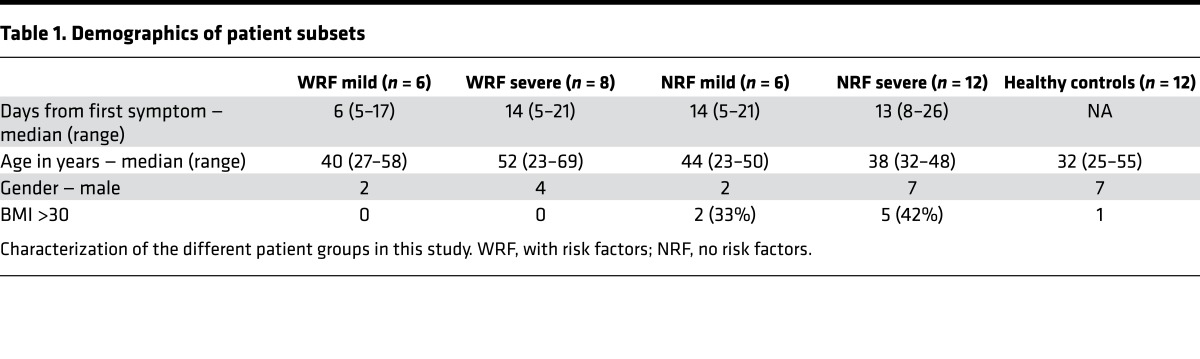
Demographics of patient subsets
