# Supplementary material for: M1-like monocytes are a major immunological determinant of severity in previously healthy adults with life-threatening influenza
Source: JCI Insight. 2017 Apr 6;2(7):e91868. doi: 10.1172/jci.insight.91868 (PMC5374077; doi:10.1172/jci.insight.91868)
Supplement: Supplemental data [file jciinsight-2-91868-s001.pdf]

Supporting information

Supplementary Figure 1 Gating strategies for cells depicted in Table 2.

Supplementary Figure 2 Viral load in humans measured at TP0 comparing **(A)** mild and severe disease in all pH1N1 patients and **(B)** comparing mild and severe NRF patients. **(C)-(D)** Viral load in the same groups as (A)-(B) shown in relation to days from illness onset. p values calculated by Mann-Whitney test in (A) and (B); and with Spearman's rank correlation in (C) and (D).

Supplementary Figure 3 **(A)-(I)** Circulating immune profile as described in Table 2, comparing mild and severe disease in NRF and WRF separately (Mann Whitney Rank Sum test if not normal distribution or Student t Test if normal distribution). **(J)** Correlation between CD14 monocytes:CD3 T cell ratio and IFN-γ Elispot SFU to pH1N1 HA [as in **(D)**] for NRF severe patients.

Supplementary Figure 4 **(A)-(B)** Circulating $CD14^+$ monocytes and $CD15^+$ LDG expressed as % of live cells for NRF (n=18) and WRF (n=14) patients. **(C)-(D)** Monocytes and LDGs at TP1 and 4-6 weeks later (TP2) for 9 patients from NRF severe group expressed as % of live cells. **(E)-(H)** Relationship between $CD14^+$ monocytes and $CD15^+$ LDG with BMI for all patients. **(I)** Difference in BMI between patients with severe and mild disease for all patients. **(J)-(K)** Data for Figure 1G-H when patients with BMI>30 are removed. All values are mean ± SEM for normally distributed sets or median ± interquartile range for non-normal distribution. p values calculated using Mann-Whitney test in non-parametric analysis and Student's t-test for parametric. Wilcoxon matched-pairs signed rank test was used for TP1 vs TP2 in (C). Correlation and signficance calculated using Spearman's rank test for (E) and

(F). p values calculated using Kuskall-Wallis test and Dunn's multiple comparison test for (A)-(B), and (J)-(K).

Supplementary Figure 5 (A) Representative TNF-α and IL-10 ICS for Figure 2H and (C)-(D) in this figure. **(B)** IL-10 expressing monocytes as % of total monocytes for NRF severe patients compared to healthy controls. **(C)-(D)** TNF-α and IL-10 expression for monocytes from patients from NRF severe group. ICS of cytokines measured following 6 hours of LPS stimulation of PBMCs and represented as proportion of $CD14^{+}$ monocytes. Paired t test used.

Supplementary Figure 6 (A) Weight change relative to initial weight in C57BL/6 mice following infection with pathogenic A/PR/8/34/H1N1 (PR8) or low pathogenic NYMC-X-179A (X-179A) influenza viruses. n=6 mice per group from 2 experiments. Difference between groups analyzed using two-way ANOVA with repeated measures on different days. Error bars are not visible for Days 2 and 3 of PR8 mice. **(B)** Viral titers in lungs of PR8- and X179A-infected mice on day 3 and 5 after inoculation. Viral titers measured by qPCR using primers for Matrix (M) gene, and normalized to b2m house keeping gene. PR8-infected mice reached limits of severity before day 5 and were culled. **(C)** Weight change following infection of 9 week old female C57BL/6 mice with 10^{4} EID50/ml PR8 that were either naïve ('PR8'), or had been pre-challenged with X179A 14 days previously ('X179A+PR8'); both groups of mice were of the same age and weight. Mice were culled at 20% weight loss limit. n=3 per group. **(D)-(E)** Number of transferred cells (identifiable as $CD45.1^{+}$ cells) at 6 and 24 hours after transfer in BAL and perfused lung digest.

Supplementary Figure 7 (A) Percentage weight change relative to initial weight following infection with either 10^{5} EID50/ml of X179A or H5N1 and uninfected mice. n=6 in each group. Difference between groups analyzed using two-way ANOVA with

repeated measures on different days. **p<0.01; ****p<0.0001. **(B)-(C)**. Viral titers in lungs of H5N1- and X179A-infected mice on day 3 and 5 after inoculation with 10^5 EID50/ml of H5N1 or X179A. Viral titers measured by qPCR using primers for Matrix (M) gene, and normalized to b2m house keeping gene. For (B), single value is combined RNA for 6 mice, which was then averaged. This was to maximize efficiency and reduce possibility of contamination in Category 4 conditions.

Supplementary Figure 8 (A-D) GSEA summaries from GSE32690, GSE53321, GSE63245 and GSE51466 showing the normalized enrichment scores of M1 or M2 gene sets in H5N1 or X179A infection on day 3 and day 5. n.s - gene set was not significantly enriched in sample. Tables show all details from GSEA analysis for all 4 gene sets analyzed. ES= enrichment score (degree to which the gene set is overrepresented at the top or bottom of the ranked input list of genes), NES= normalized enrichment score (adjusted for gene set size or multiple hypothesis testing), NOM P= nominal p value, FDR q= false discovery rate, FWER Q= family wise-error rate, size= number of genes in analysis after filtering out those not in expression dataset.

Supplementary Table 1. Characterization of previously healthy pH1N1-infected patients with no risk factors (NRF) for severe disease. 'Resp score' – respiratory score as defined in Methods.

Supplementary Table 2 Gene ontology terms that were enriched in H5N1 or X179A infection on day 3 and day 5, analyzed by GOrilla. Only GO terms with at least 5 genes ('b') were used in the analysis. Enrichment of these terms was tested against gene lists ranked in order of highest to lowest expression in infected mice (H5N1 or X-179A) relative to uninfected controls. Enrichment = (b/n) / (B/N): N- the total number of genes; B- the total number of genes associated with a specific GO

term; n- the number of genes in the top of the user's input list; b- the number of genes in the intersection.

Supplementary Table 3 Gene sets used to identify M1 or M2 macrophage genes for use in GSEA for GSEA (maximum input).

Supplementary Table 4 Antibodies used in mouse flow cytometry analysis.

Supplementary Table 5 Antibodies used in human flow cytometry analysis.

Supplementary Table 6 qPCR primers used for human monocyte M1-M2 gene expression studies and mouse viral load assessment.

Supplementary Table 7 Top genes significantly upregulated in M1 or M2 macrophages at adjusted p<0.05 and logFC>1.5. Gene sets from published work were analysed using GEO2R and used for input into GSEA.

MOSAIC data and clinical samples were provided by the MOSAIC consortium.****MOSAIC investigators: Chelsea and Westminster NHS Foundation Trust:** B.G. Gazzard**. Francis Crick Institute, Mill Hill Laboratory:** A. Hay, J. McCauley, A. O'GGarra. **Imperial College London, UK:** P. Aylin, D. Ashby, W.S. Barclay, S.J. Brett, W.O. Cookson, M.J. Cox, J. Dunning, L.N. Drumright, R.A. Elderfield, L. Garcia-Alvarez, M.J. Griffiths, M.S. Habibi, T.T. Hansel, J.A. Herberg, A.H. Holmes, S.L. Johnston, O.M. Kon, M. Levin, M.F. Moffatt, S. Nadel, J.O. Warner. **Liverpool School of Tropical Medicine, UK:** S.J. Aston, S.B. Gordon. **Manchester Collaborative Centre for Inflammation Research (MCCIR)** T. Hussell. **Public Health England (formerly Health Protection Agency), UK:** C.

Thompson, M.C. Zambon. **The Roslin Institute, University of Edinburgh:** D.A. Hume. **University College London, UK**: A. Hayward. **UCL Institute of Child Health:** R.L. Smyth; **University of Edinburgh, UK:** J.K. Baillie, P. Simmonds **University of Liverpool, UK:** P.S. McNamara; M.G. Semple; **University of Nottingham, UK**: J.S. Nguyen-Van-Tam; **University of Oxford, UK:** L-P. Ho, A. J. McMichael **Wellcome Trust Sanger Institute, UK:** P. Kellam **West of Scotland Specialist Virology Centre, Glasgow, UK:** W.E. Adamson, W.F. Carman.

Supplemental Figure 1

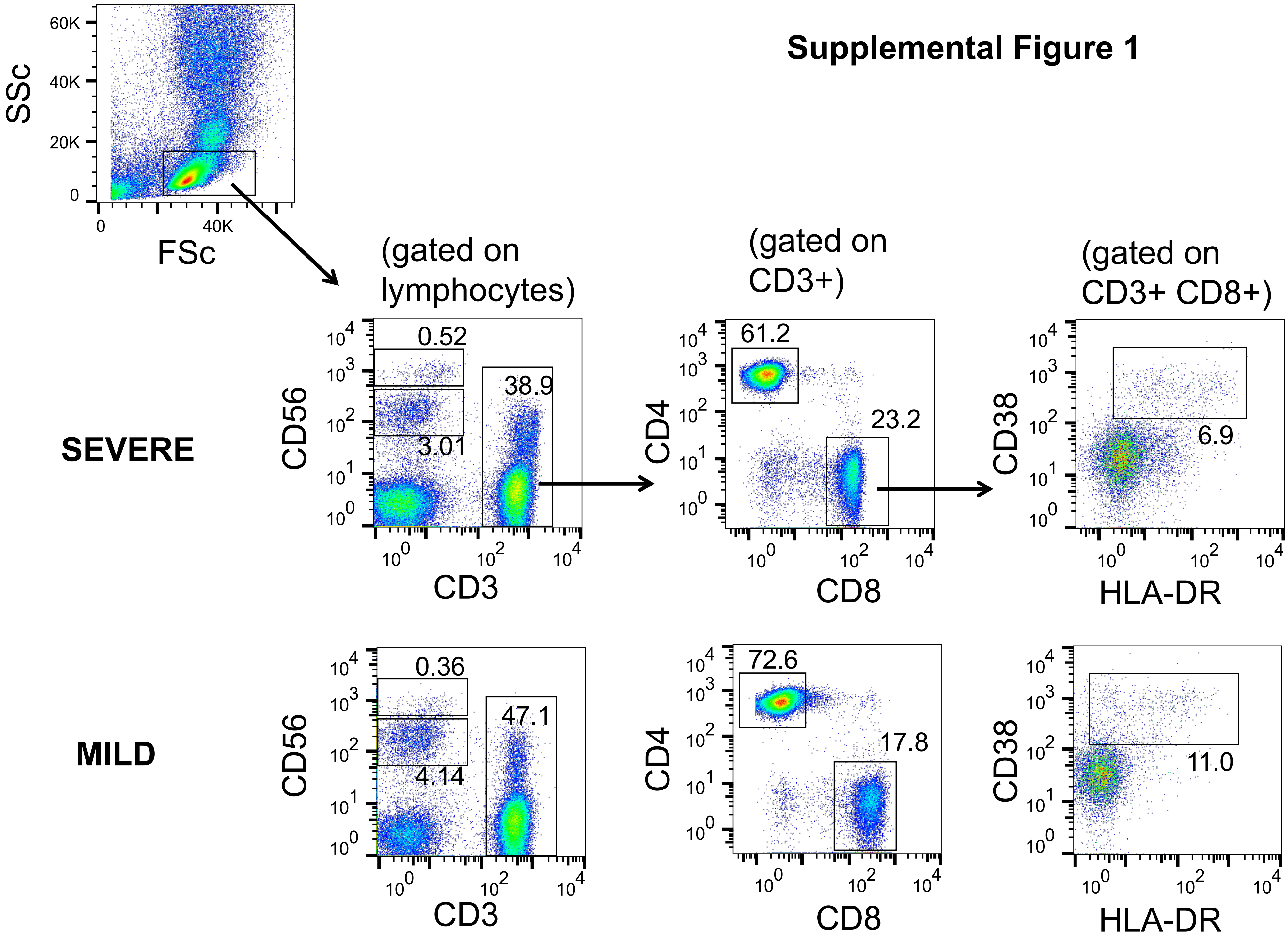

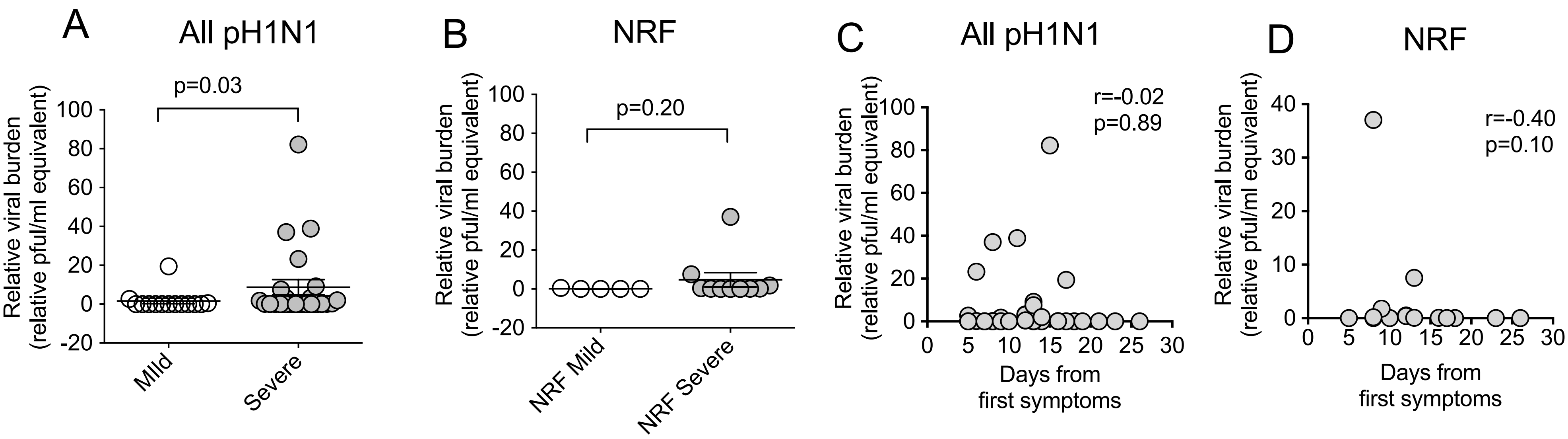

Supplemental Figure 2

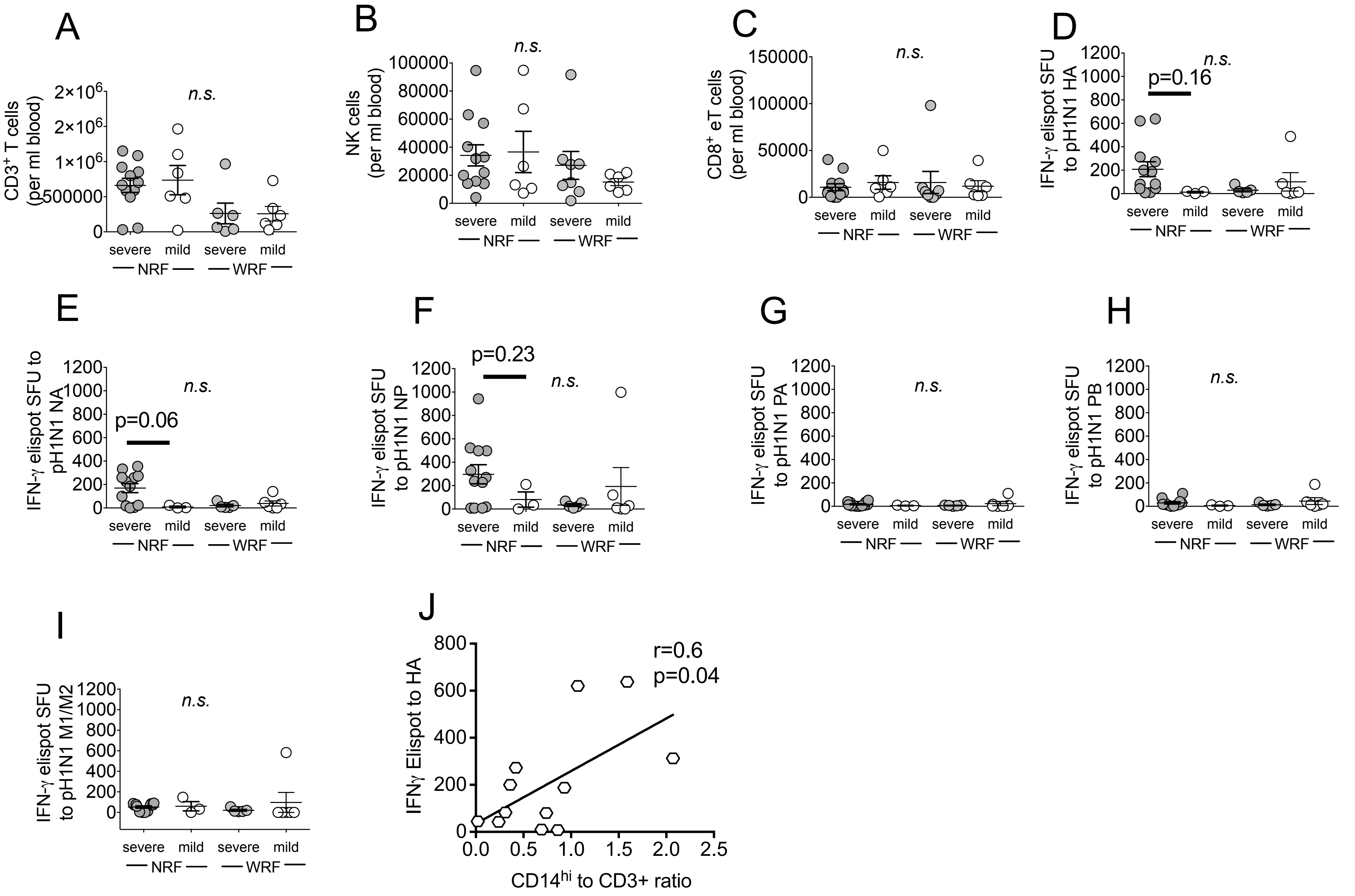

Supplemental Figure 3

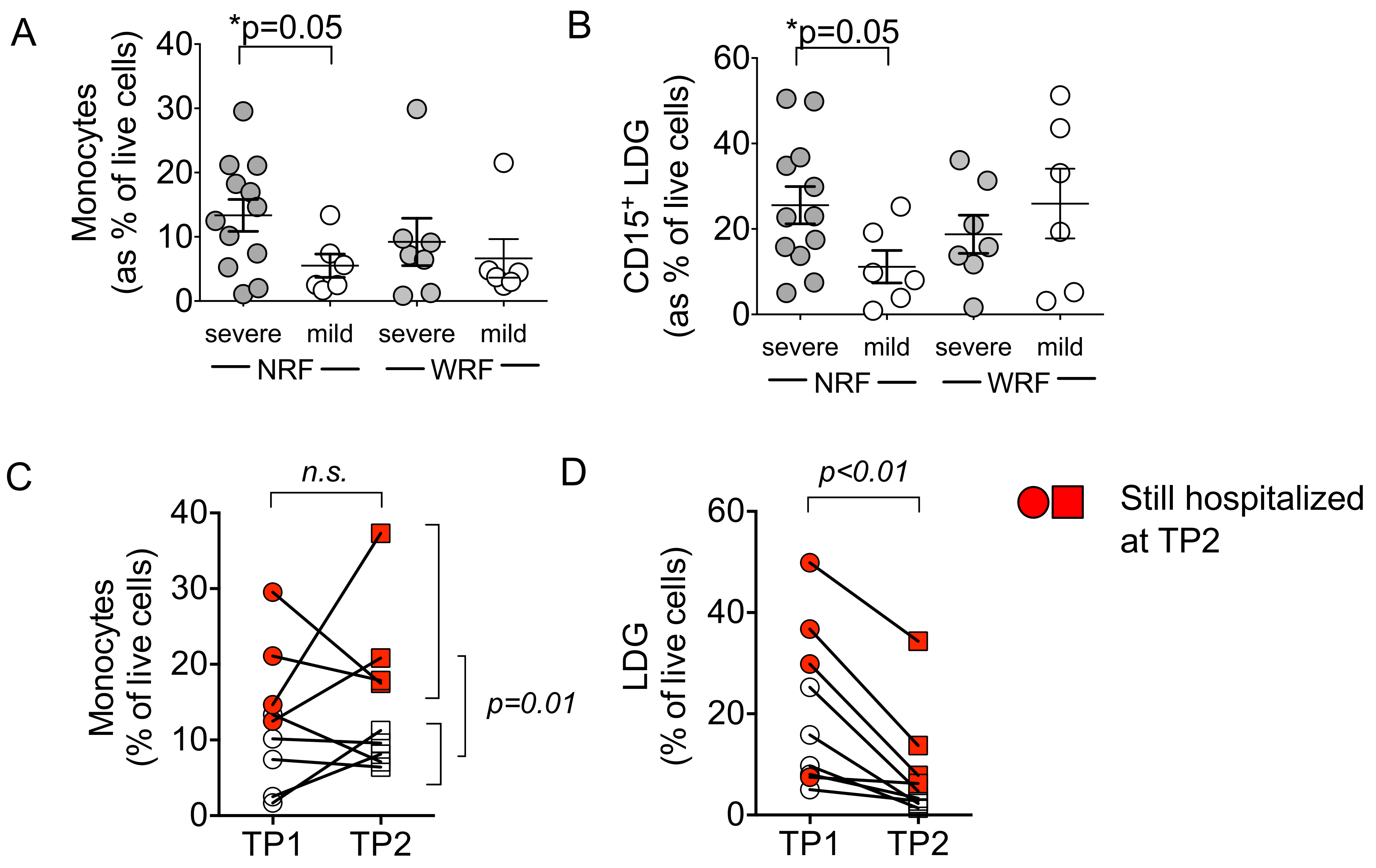

Supplemental Figure 4

Supplemental Figure 4

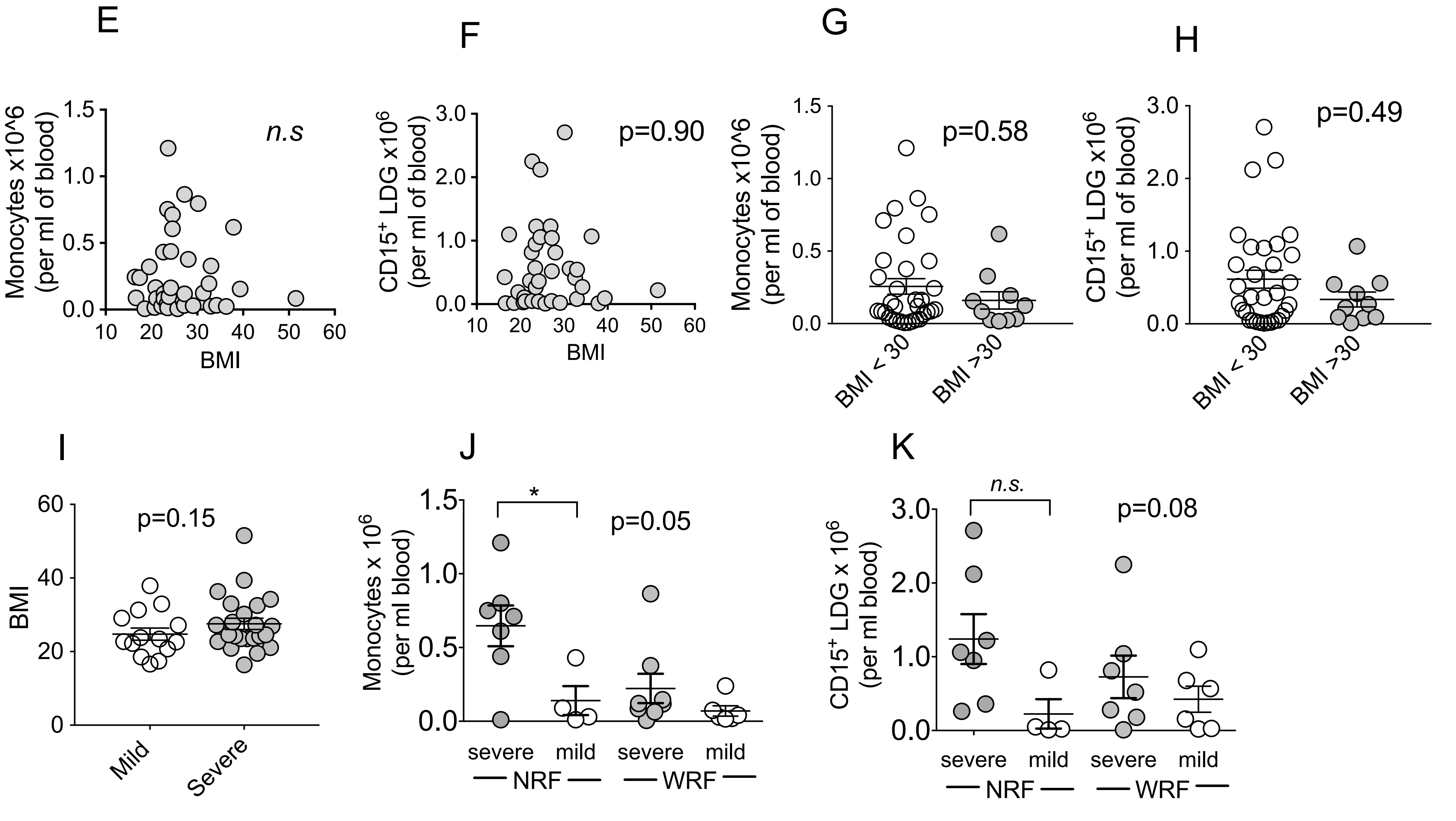

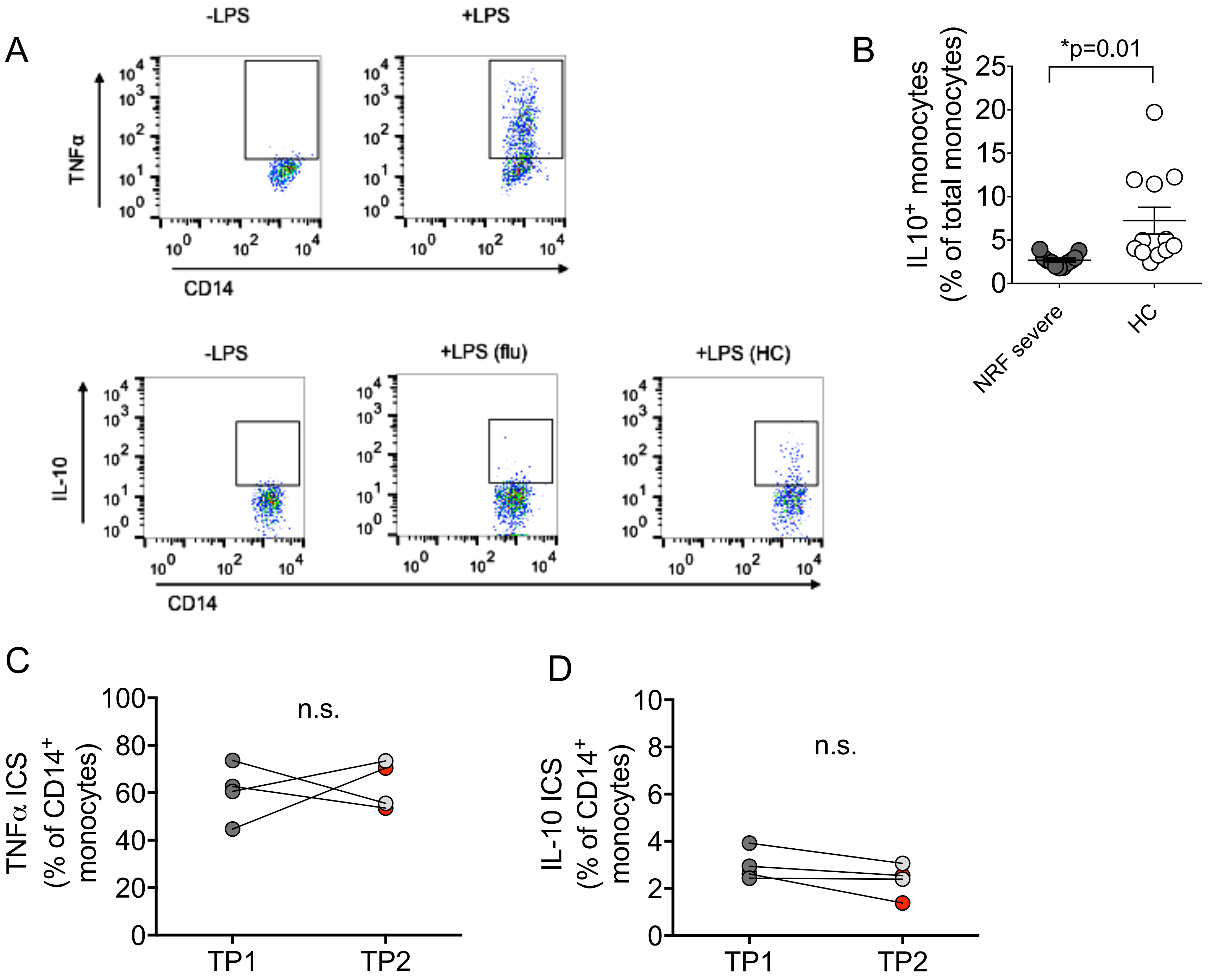

Supplemental Figure 5

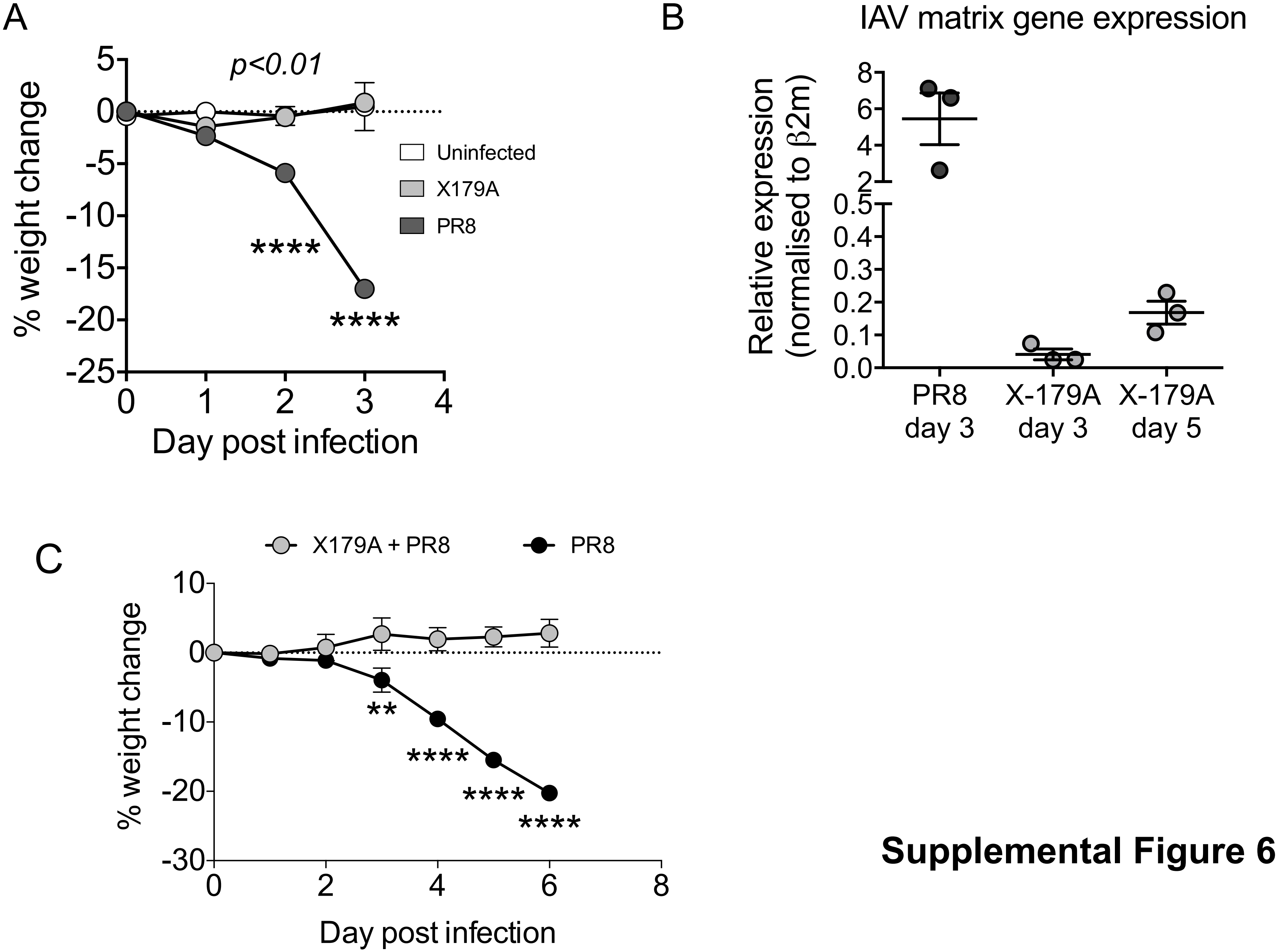

Supplemental Figure 6

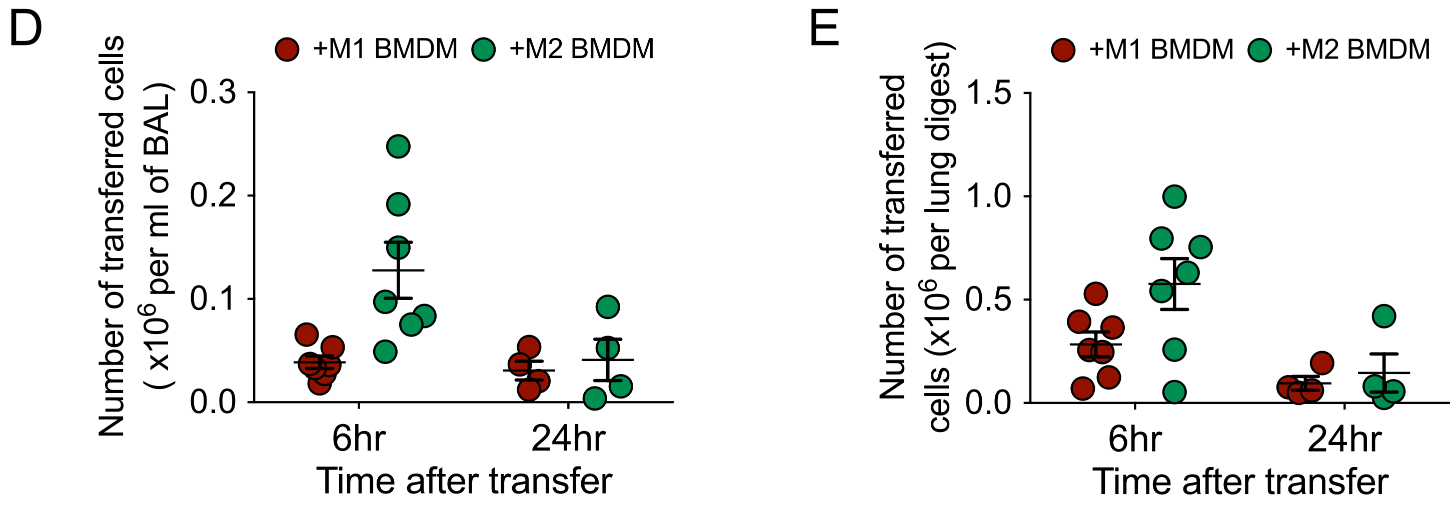

Supplemental Figure 6

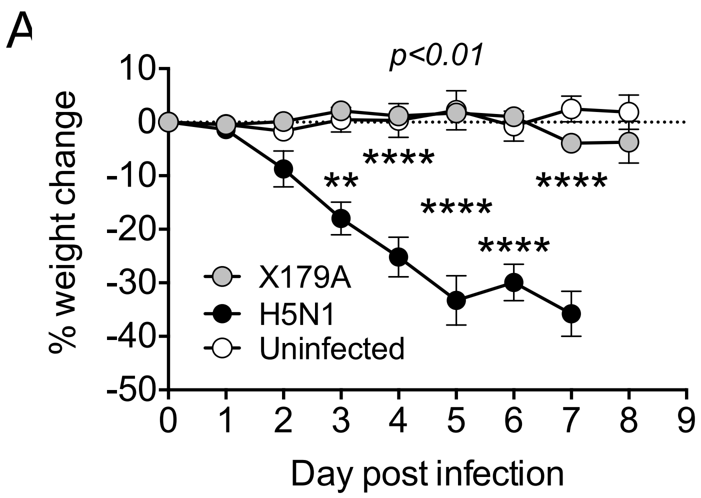

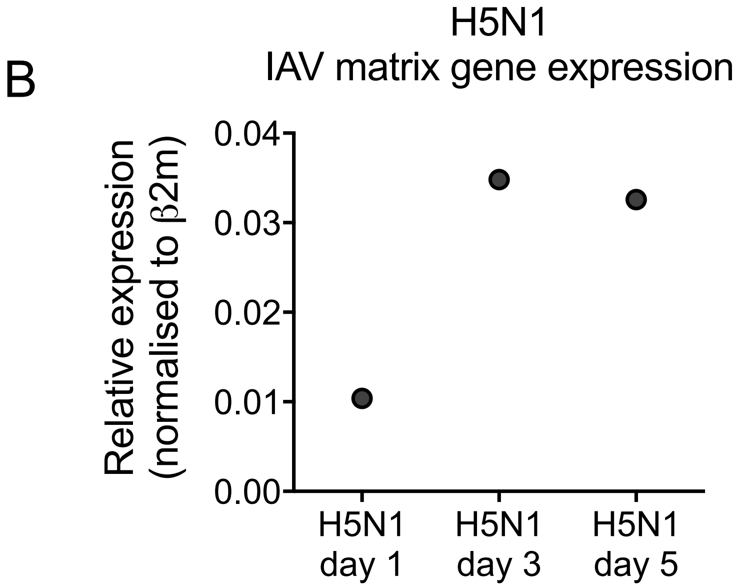

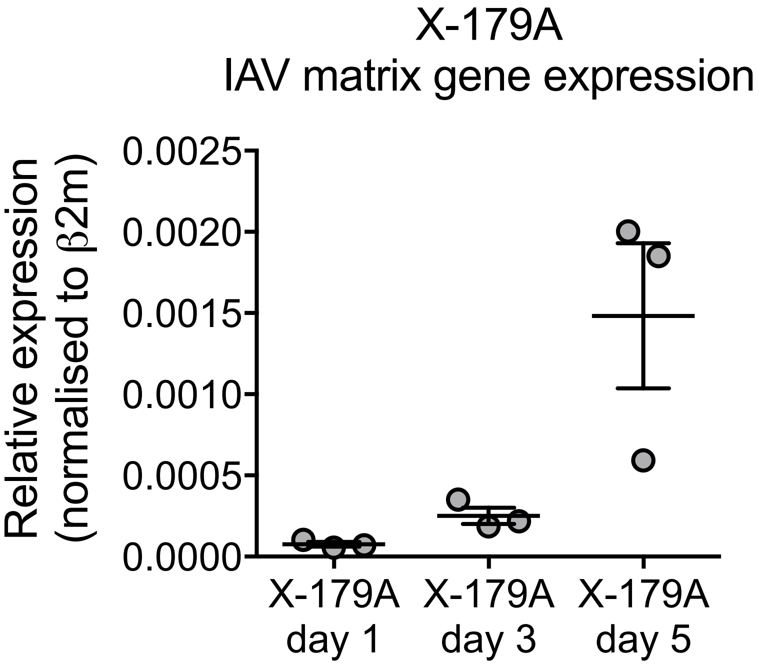

Supplemental figure 7

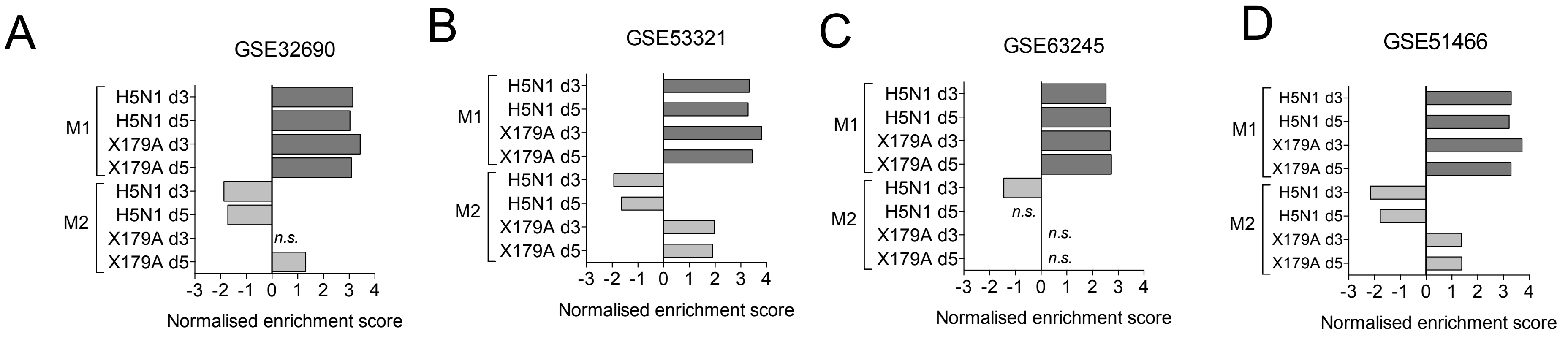

E

M1 enrichment

		ES	NES	NOM P	FDR Q	FWER Q	SIZE
H5N1 day 3	GSE53321	0.84365904	3.328533	<0.001	<0.001	<0.001	341
	GSE32690	0.790956	3.141693	<0.001	<0.001	<0.001	377
	GSE63245	0.67052794	2.526454	<0.001	<0.001	<0.001	222
	GSE51466	0.8343884	3.3040514	<0.001	<0.001	<0.001	350
H5N1 day 5	GSE53321	0.84162766	3.2827406	<0.001	<0.001	<0.001	341
	GSE32690	0.7722635	3.0297759	<0.001	<0.001	<0.001	377
	GSE63245	0.60343194	2.688004	<0.001	<0.001	<0.001	222
	GSE51466	0.8257437	3.2191389	<0.001	<0.001	<0.001	350
X179A day 3	GSE53321	0.8291504	3.8154461	<0.001	<0.001	<0.001	341
	GSE32690	0.7336535	3.4319682	<0.001	<0.001	<0.001	377
	GSE63245	0.60343194	2.688004	<0.001	<0.001	<0.001	222
	GSE51466	0.79974025	3.7220237	<0.001	<0.001	<0.001	350
X179A day 5	GSE53321	0.85203594	3.4437249	<0.001	<0.001	<0.001	341
	GSE32690	0.76370096	3.0890503	<0.001	<0.001	<0.001	377
	GSE63245	0.7066271	2.7333457	<0.001	<0.001	<0.001	222
	GSE51466	0.824262	3.294722	<0.001	<0.001	<0.001	350

F

M2 enrichment

		ES	NES	NOM P	FDR Q	FWER Q	SIZE
H5N1 day 3	GSE53321	-0.42260036	-1.9371371	<0.001	<0.001	0.011	345
	GSE32690	-0.40993422	-1.8679197	<0.001	<0.001	<0.001	389
	GSE63245	-0.39475	-1.4463	0.02162	0.01544	0.008	76
	GSE51466	-0.49340945	-2.1631439	<0.001	<0.001	<0.001	230
H5N1 day 5	GSE53321	-0.35845533	-1.638027	<0.001	<0.001	0.092	345
	GSE32690	-0.3730197	-1.7139028	<0.001	<0.001	<0.001	389
	GSE63245	0.24507721	0.90729165	0.60628	0.64588	0.589	76
	GSE51466	-0.41163298	-1.7746121	<0.001	<0.001	<0.001	230
X179A day 3	GSE53321	0.42457503	1.9630932	<0.001	<0.001	<0.001	345
	GSE32690	0.2422696	1.1387417	0.15213	0.15668	0.197	389
	GSE63245	0.24507721	0.90729165	0.60628	0.64588	0.589	76
	GSE51466	0.30876794	1.374535	0.01774	0.02013	0.024	230
X179A day 5	GSE53321	0.47212347	1.9041343	<0.001	<0.001	<0.001	345
	GSE32690	0.32565087	1.3115122	0.02813	0.02520	0.048	389
	GSE63245	0.24529232	0.8171897	0.75915	0.80147	0.856	76
	GSE51466	0.35663462	1.3872733	0.02539	0.01502	0.019	230

Supplemental figure 8

Patient number (NRF)	TP2	Resp score	BMI	Mild (M) or severe (S)	Comorbidities	Days from first symptom	Age	Gender
1	yes	1	33	M	NONE	5	50	F
2		3	24	S	NONE	9	33	F
3	yes	1	>30	M	NONE	8	43	M
4		1	27	M	NONE	18	45	F
5		3	24	S	NONE	12	40	M
6		3	52	S	NONE	16	48	F
7		3	36	S	NONE	8	36	F
8		2	23	S	NONE	10	33	F
9	yes	3	24	S	NONE	26	35	M
10		1	19	M	NONE	10	23	M
11	yes	3	39	S	NONE	8	44	M
12		3	25	S	NONE	15	32	M
13	yes	3	25	S	NONE	13	43	M
14		3	30	S	NONE	13	44	M
15		1	17	M	NONE	12	50	F
16	yes	3	33	S	NONE	17	32	M
17	yes	3	33	S	NONE	23	42	F
18	yes	1	23	M	NONE	10	44	F

Supplemental table 1

		H5N1 day 3			H5N1 day 5		
		P-value	FDR q-value	Enrichment (N, B, n, b)	P-value	FDR q-value	Enrichment (N, B, n, b)
GO:0090026	positive regulation of monocyte chemotaxis	4.22E-08	1.64E-06	**34.22** (16186,11,258,6)	6.18E-09	2.66E-07	**27.91** (16186,11,369,7)
GO:0090025	regulation of monocyte chemotaxis	9.04E-08	3.27E-06	**31.37** (16186,12,258,6)	1.50E-08	6.05E-07	**25.59** (16186,12,369,7)
GO:0042116	macrophage activation	2.53E-08	1.04E-06	**8.37** (16186,24,967,12)	5.40E-08	2.02E-06	**11.24** (16186,24,600,10)
GO:0010758	regulation of macrophage chemotaxis	3.56E-06	9.99E-05	**6.13** (16186,16,1651,10)	8.92E-05	1.80E-03	**6.09** (16186,16,1328,8)
GO:0045649	regulation of macrophage differentiation	3.37E-04	5.78E-03	**7.40** (16186,13,1009,6)	1.54E-04	2.90E-03	**8.60** (16186,13,869,6)
GO:0071621	granulocyte chemotaxis	3.33E-17	5.50E-15	**17.94** (16186,41,396,18)	3.03E-18	5.51E-16	**13.91** (16186,41,596,21)
GO:0071622	regulation of granulocyte chemotaxis	1.34E-12	1.08E-10	**8.49** (16186,32,1072,18)	5.45E-09	2.37E-07	**10.47** (16186,32,580,12)
GO:0097530	granulocyte migration	1.01E-16	1.52E-14	**17.11** (16186,43,396,18)	1.16E-17	2.02E-15	**13.26** (16186,43,596,21)
GO:0050870	positive regulation of T cell activation	2.35E-08	9.80E-07	**4.86** (16186,102,685,21)	1.75E-09	7.98E-08	**4.06** (16186,102,1094,28)
GO:0042110	T cell activation	5.34E-11	3.45E-09	**3.07** (16186,160,1449,44)	2.30E-11	1.43E-09	**2.93** (16186,160,1657,48)
GO:0042129	regulation of T cell proliferation	8.08E-09	3.75E-07	**5.19** (16186,106,618,21)	1.07E-09	5.02E-08	**5.81** (16186,106,552,21)
GO:0070489	T cell aggregation	6.62E-11	4.14E-09	**3.05** (16186,161,1449,44)	2.75E-11	1.67E-09	**2.91** (16186,161,1657,48)
GO:0046631	alpha-beta T cell activation	4.55E-07	1.44E-05	**4.43** (16186,39,1591,17)	4.74E-08	1.79E-06	**5.66** (16186,39,1174,16)
GO:0042102	positive regulation of T cell proliferation	3.01E-07	9.77E-06	**6.40** (16186,60,590,14)	4.08E-08	1.56E-06	**7.55** (16186,60,500,14)
GO:0001914	regulation of T cell mediated cytotoxicity	7.00E-05	1.45E-03	**6.30** (16186,16,1284,8)	5.62E-05	1.20E-03	**8.21** (16186,16,863,7)
GO:2000404	regulation of T cell migration	4.71E-07	1.48E-05	**5.54** (16186,23,1650,13)	1.11E-08	4.60E-07	**10.63** (16186,23,728,11)
GO:0072678	T cell migration	1.82E-08	7.79E-07	**11.26** (16186,11,1176,9)	2.35E-07	7.96E-06	**11.35** (16186,11,1037,8)
GO:0045580	regulation of T cell differentiation	1.74E-04	3.26E-03	**3.80** (16186,87,685,14)	5.46E-04	8.68E-03	**2.61** (16186,87,1425,20)
GO:0045582	positive regulation of T cell differentiation	1.37E-05	3.41E-04	**5.45** (16186,52,685,12)	1.11E-04	2.18E-03	**7.39** (16186,52,337,8)
GO:0046635	positive regulation of alpha-beta T cell activation	9.28E-04	1.40E-02	**5.11** (16186,37,685,8)	8.65E-04	1.26E-02	**6.12** (16186,37,500,7)
GO:0050863	regulation of T cell activation	1.83E-10	1.11E-08	**4.05** (16186,181,685,31)	5.43E-14	5.15E-12	**3.26** (16186,181,1428,52)
GO:0019724	B cell mediated immunity	5.08E-08	1.94E-06	**6.41** (16186,21,1562,13)	1.15E-06	3.40E-05	**6.52** (16186,21,1300,11)
GO:0002712	regulation of B cell mediated immunity	1.18E-07	4.14E-06	**5.42** (16186,30,1494,15)	2.54E-06	7.15E-05	**11.54** (16186,30,374,8)
GO:0002714	positive regulation of B cell mediated immunity	1.55E-07	5.34E-06	**6.19** (16186,24,1416,13)	6.56E-06	1.66E-04	**12.62** (16186,24,374,7)
GO:0002715	regulation of natural killer cell mediated immunity	8.36E-06	2.15E-04	**4.88** (16186,24,1657,12)	1.20E-07	4.25E-06	**8.60** (16186,24,863,11)
GO:0042269	regulation of natural killer cell mediated cytotoxicity	8.36E-06	2.15E-04	**4.88** (16186,24,1657,12)	1.20E-07	4.24E-06	**8.60** (16186,24,863,11)

Supplemental table 2

		X179A day 3			X179A day 5		
		P-value	FDR q-value	Enrichment (N, B, n, b)	P-value	FDR q-value	Enrichment (N, B, n, b)
GO:0090026	positive regulation of monocyte chemotaxis	1.17E-05	6.59E-04	**13.06** (16186,11,676,6)	2.18E-09	1.38E-07	**32.80** (16186,11,314,7)
GO:0090025	regulation of monocyte chemotaxis	2.29E-05	1.17E-03	**11.97** (16186,12,676,6)	5.01E-09	2.98E-07	**30.07** (16186,12,314,7)
GO:0042116	macrophage activation	4.51E-05	2.08E-03	**7.40** (16186,24,729,8)	5.16E-06	1.75E-04	**10.05** (16186,24,537,8)
GO:0010758	regulation of macrophage chemotaxis	4.09E-04	1.21E-02	**10.58** (16186,16,478,5)	7.34E-05	1.70E-03	**10.52** (16186,16,577,6)
GO:0045649	regulation of macrophage differentiation	2.45E-04	8.05E-03	**7.88** (16186,13,948,6)	2.90E-04	5.45E-03	**10.79** (16186,13,577,5)
GO:0071621	granulocyte chemotaxis	3.29E-04	1.02E-02	**6.12** (16186,41,516,8)	3.33E-10	2.34E-08	**10.31** (16186,41,536,14)
GO:0071622	regulation of granulocyte chemotaxis	4.00E-04	1.18E-02	**11.99** (16186,32,211,5)	3.27E-07	1.41E-05	**8.37** (16186,32,665,11)
GO:0097530	granulocyte migration	4.53E-04	1.29E-02	**5.84** (16186,43,516,8)	7.47E-10	4.94E-08	**9.83** (16186,43,536,14)
GO:0050870	positive regulation of T cell activation	3.21E-10	5.91E-08	**3.47** (16186,102,1600,35)	9.05E-14	1.19E-11	**5.22** (16186,102,1004,33)
GO:0042110	T cell activation	3.40E-04	1.04E-02	**2.80** (16186,160,651,18)	6.95E-18	1.70E-15	**3.84** (16186,160,1500,57)
GO:0042129	regulation of T cell proliferation	6.66E-08	6.36E-06	**3.08** (16186,106,1588,32)	1.41E-11	1.20E-09	**5.02** (16186,106,852,28)
GO:0070489	T cell aggregation	3.67E-04	1.11E-02	**2.78** (16186,161,651,18)	9.87E-18	2.37E-15	**3.82** (16186,161,1500,57)
GO:0046631	alpha-beta T cell activation	9.99E-04	2.40E-02	**5.10** (16186,39,651,8)	3.02E-11	2.44E-09	**5.81** (16186,39,1500,21)
GO:0042102	positive regulation of T cell proliferation	1.67E-07	1.49E-05	**4.04** (16186,60,1401,21)	4.36E-10	2.96E-08	**5.64** (16186,60,1004,21)
GO:0001914	regulation of T cell mediated cytotoxicity	3.02E-06	2.02E-04	**12.88** (16186,16,550,7)	6.17E-07	2.55E-05	**11.95** (16186,16,677,8)
GO:2000404	regulation of T cell migration	7.13E-05	3.01E-03	**16.68** (16186,23,211,5)	1.23E-06	4.76E-05	**23.59** (16186,23,179,6)
GO:0072678	T cell migration	3.20E-05	1.55E-03	**10.77** (16186,11,820,6)	2.14E-08	1.11E-06	**11.04** (16186,11,1200,9)
GO:0045580	regulation of T cell differentiation	7.26E-05	3.04E-03	**4.13** (16186,87,630,14)	4.19E-07	1.77E-05	**4.04** (16186,87,966,21)
GO:0045582	positive regulation of T cell differentiation	2.36E-05	1.19E-03	**5.16** (16186,52,724,12)	1.31E-06	5.02E-05	**4.37** (16186,52,1210,17)
GO:0046635	positive regulation of alpha-beta T cell activation	1.90E-04	6.64E-03	**6.50** (16186,37,538,8)	6.15E-05	1.47E-03	**4.47** (16186,37,1175,12)
GO:0050863	regulation of T cell activation	8.45E-09	9.51E-07	**2.57** (16186,181,1600,46)	1.46E-14	2.47E-12	**4.10** (16186,181,1004,46)
GO:0019724	B cell mediated immunity	2.72E-06	1.86E-04	**10.61** (16186,21,581,8)	1.55E-06	5.80E-05	**11.50** (16186,21,536,8)
GO:0002712	regulation of B cell mediated immunity	1.71E-05	9.05E-04	**4.36** (16186,30,1608,13)	2.24E-07	1.01E-05	**5.72** (16186,30,1321,14)
GO:0002714	positive regulation of B cell mediated immunity	3.54E-05	1.71E-03	**9.67** (16186,24,488,7)	7.34E-06	2.40E-04	**5.62** (16186,24,1321,11)
GO:0002715	regulation of natural killer cell mediated immunity	7.78E-05	3.23E-03	**4.39** (16186,24,1689,11)	3.00E-04	5.60E-03	**6.87** (16186,24,687,7)
GO:0042269	regulation of natural killer cell mediated cytotoxicity	7.78E-05	3.22E-03	**4.39** (16186,24,1689,11)	3.00E-04	5.60E-03	**6.87** (16186,24,687,7)

Supplemental table 2 cont

	Title	Macrophage treatment for M1 or M2 (n=)	Citation
GSE53321	Expression data from polarized macrophages: effect of p53	Bone marrow derived macrophages polarised with E.coli LPS+IFN-γ (M1) or IL4+IL13 (M2)	Li L, Ng DS, Mah WC, Almeida FF et al. A unique role for p53 in the regulation of M2 macrophage polarization. Cell Death Differ 2015 Jul;22(7):1081-93. PMID: 25526089
GSE32690	IFN-γ-induced iNOS expression in regulatory macrophages prolongs allograft graft survival in fully immunocompetent recipients	FACS sorted L6C+ Ly6G- CD11b+ monocytes from bone marrow cultured for 6 days in 10% FCS + 20ng/ml M-CSF, then for 24 hours with 25ng/ml IFN-g + 100ug/ml LPS (M1) or 20ng/ml IL-4 (M2)	Riquelme P, Tomiuk S, Kammler A, Fändrich F et al. IFN-γ-induced iNOS expression in mouse regulatory macrophages prolongs allograft survival in fully immunocompetent recipients. Mol Ther 2013 Feb;21(2):409-22. PMID: 22929659
GSE63245	Gene expression data from murine M1 and M2 macrophages	Bone marrow derived cells cultured with G-MCSF (M1) or M-CSF (M2) for 7 days	n/a
GSE51466	Polarization profiling of renal macrophages in stone model mice	Bone marrow derived macrophages grown in L-conditioned medium over 7 days. BMM were stimulated for 20 hours with 100ng/ml LPS and 20ng/ml GM-CSF (M1) or 50ng/ml IL-4 and 10ng/ml M-CSF (M2)	Taguchi K, Okada A, Kitamura H, Yasui T et al. Colony-stimulating factor-1 signaling suppresses renal crystal formation. J Am Soc Nephrol 2014 Aug;25(8):1680-97. PMID: 24578130

Supplemental table 3

Antibody	Conjugate	Clone	Host & Isotype	Supplier	Cat. Number
CCR2	APC	475301	Rat IgG2B	R&D	FAB5538A
CCR7	eFluor450	4B12	Rat IgG2a, κ	eBioscience	48-1971
CD11c	PE	N418	Hamster IgG	Miltenyi	130-102-799
CD206	PE-cy7	C068C2	Rat IgG2a, κ	BioLegend	141719
CD3	APC-eFluor780	145-2C11	Armenian Hamster IgG	eBioscience	47-0031
CD86	FITC	GL1	Rat IgG2a, κ	eBioscience	11-0862
Dectin-1	PE	RH1	Rat IgG1, κ	BioLegend	144303
F4/80	APC-cy7	BM8	Rat IgG2a, κ	BioLegend	123118
Ly6C	eFluor450	HK1.4	Rat IgG2c, κ	eBioscience	48-5932
Ly6G	FITC	1A8	Rat IgG2A	Miltenyi	130-093-138
Ly6G	APC	1A8	Rat IgG2a, κ	BioLegend	127614
Zombie Aqua™	BV510	n/a	n/a	BioLegend	423101

Supplementary table 4

Antibody	Conjugate	Clone	Host & Isotype	Supplier	Cat. Number
CCR7	PB	G043H7	Mouse IgG2a, κ	BioLegend	353210
CD11b	APCe-fluor780	ICRF44	Mouse IgG1, κ	eBioscience	47-0118
CD14	PEcy7	61D3	Mouse IgG1, κ	eBioscience	25-0149
CD15	APC	HI98	Mouse IgM, κ	BioLegend	301907
CD16	eFluor450	CB16	Mouse IgG1, κ	eBioscience	48-0168
CD16	PE	B73.1	Mouse IgG1, κ	BioLegend	360704
CD163	PE	GHI/61	Mouse IgG1, κ	BioLegend	333606
CD3	APC-eFluor780	UCHT1	Mouse IgG1, κ	eBioscience	47-0038
CD3	FITC	UCHT1	Mouse IgG1, κ	eBioscience	11-0038
CD38	APC	HIT2	Mouse IgG1, κ	eBioscience	17-0389
CD4	PB	RPA-T4	Mouse IgG1, κ	BioLegend	300521
CD4	PEcy7	RPA-T4	Mouse IgG1, κ	eBioscience	25-0049
CD56	PEcy7	HCD56	Mouse IgG1, κ	BioLegend	318318
CD8a	eFluor450	RPA-T8	Mouse IgG1, κ	eBioscience	48-0088
HLA-DR	FITC	L243	Mouse IgG2a, κ	BioLegend	307603
Vα24 Jα18	FITC	6B11	Mouse IgG1, κ	BD	558371
IL-10	APC	JES3-9D7	Rat IgG1, κ	BioLegend	501410
IL-6	FITC	MQ2-13A5	Rat IgG1, κ	BioLegend	501104
TNFα	eFluor450	Mab11	Mouse IgG1, κ	eBioscience	48-7349
Fixable viability dye	eFluor780	n/a	n/a	eBioscience	65-0865

Supplementary table 5

Gene symbol	Gene Description	Forward primer	Reverse primer
ACTB	Actin, beta (ACTB)	CCTGGCACCCAGCACAAT	GCCGATCCACACGGAGTACT
CD163	CD163 molecule	TTTGTCAACTTGAGTCCCTTCAC	TCCCGCTACACTTGTTTTCAC
CD200R1	CD200 receptor 1	GACCAGAGAGGGTCTCACCA	TTGAAGCGGCCACTAAGAAG
GBP1	Guanylate binding protein 1, interferon-inducible	AGGAGTTCCTTCAAAGATGTGGA	GCAACTGGACCCTGTCGTT
IDO1	Indoleamine 2,3-dioxygenase 1 (IDO1)	GCCAGCTTCGAGAAAGAGTTG	ATCCCAGAACTAGACGTGCAA
IL-8	Interleukin 8	TGCTAAAGAACTTAGATGTCAGTGCAT	TGGTCCACTCTCAATCACTCTCA
IL10	Interleukin 10	TACGGCGCTGTCATCGATT	GGCTTTGTAGATGCCTTTCTCTTG
IL12b	Interleukin 12B (p40)	AGAAGATGGTATCACCTGGACC	GAACCTCGCCTCCTTTGTGAC
IL6	Interleukin 6 (interferon, beta 2)	AGTAACATGTGTGAAAGCAGCAAAG	GGCAAGTCTCCTCATTGAATCC
MRC1	mannose receptor, C type 1 (MRC1)	AAGGCGGTGACCTCACAAG	AAAGTCCAATTCCTCGATGGTG
TGM2	transglutaminase 2 (TGM2)	GCCACTTCATTTTGCTCTTCAA	TCCTCTTCCGAGTCCAGGTACA
TNFalpha	Tumor necrosis factor	GGCCAAGCCCTGGTATGAG	TAGTCGGGCCGATTGATCTC

Primer	Forward primer	Reverse primer
Influenza Matrix	CTTCTAACCGAGGTCGAAACGTA	GGTGACAGGATTGGTCTTGTCTTTA

Supplementary table 6
